# Enhanced Costimulatory Signaling Improves CAR T-cell Effector Responses in CLL

**DOI:** 10.1158/2767-9764.CRC-22-0200

**Published:** 2022-09-30

**Authors:** McKensie A. Collins, In-Young Jung, Ziran Zhao, Kimberly Apodaca, Weimin Kong, Stefan Lundh, Joseph A. Fraietta, Arnon P. Kater, Clare Sun, Adrian Wiestner, J. Joseph Melenhorst

**Affiliations:** 1 Department of Pathology and Laboratory Medicine, Perelman School of Medicine, University of Pennsylvania, Philadelphia, Pennsylvania.; 2 Center for Cellular Immunotherapies, Perelman School of Medicine, University of Pennsylvania, Philadelphia, Pennsylvania.; 3 Parker Institute for Cancer Immunotherapy, Perelman School of Medicine, University of Pennsylvania, Philadelphia, Pennsylvania.; 4 Department of Microbiology, Perelman School of Medicine, University of Pennsylvania, Philadelphia, Pennsylvania.; 5 Abramson Cancer Center, Perelman School of Medicine, University of Pennsylvania, Philadelphia, Pennsylvania.; 6 Amsterdam UMC, University of Amsterdam, Department of Hematology, Cancer Center Amsterdam, Lymphoma and Myeloma Center Amsterdam, Amsterdam, the Netherlabds.; 7 National Heart, Lung, and Blood Institute, NIH, Bethesda, Maryland.

## Abstract

CD19-redirected chimeric antigen receptor (CAR) T cells have shown remarkable activity against B-cell cancers. While second-generation CARs induce complete remission in >80% of patients with acute lymphoblastic leukemia, similar monotherapy induces long-term remissions in only 26% of patients with chronic lymphocytic leukemia (CLL). This disparity is attributed to cell-intrinsic effector defects in autologous CLL-derived T cells. However, the mechanisms by which leukemic cells impact CAR T-cell potency are poorly understood. Herein we describe an *in vitro* assay that recapitulates endogenous CLL-mediated T-cell defects in healthy donor CAR T cells. Contact with CLL cells insufficiently activates, but does not irreversibly impair, CAR T-cell function. This state is rescuable by strong antigenic stimulation or IL2, and is not driven by immune suppression. Rather, this activation defect is attributable to low levels of costimulatory molecules on CLL cells, and exogenous costimulation enhanced CAR T-cell activation. We next assessed the stimulatory phenotype of CLL cells derived from different niches within the same patient. Lymph node (LN)-derived CLL cells had a strong costimulatory phenotype and promoted better CAR T-cell degranulation and cytokine production than matched peripheral blood CLL cells. Finally, *in vitro* CD40L-activated CLL cells acquired a costimulatory phenotype similar to the LN-derived tumor and stimulated improved CAR T-cell proliferation, cytokine production, and cytotoxicity. Together, these data identify insufficient activation as a driver of poor CAR T-cell responses in CLL. The costimulatory phenotype of CLL cells drives differential CAR T-cell responses, and can be augmented by improving costimulatory signaling.

**Significance:**

CLL cells insufficiently activate CAR T cells, driven by low levels of costimulatory molecules on the tumor. LN-derived CLL cells are more costimulatory and mediate enhanced CAR T-cell killing. This costimulatory phenotype can be modeled via CD40 L activation, and the activated tumor promotes stronger CAR T-cell responses.

## Introduction

Chronic lymphocytic leukemia (CLL) is a mature B-cell malignancy that accounts for nearly one-third of adult leukemia diagnoses in the West (1). Standard-of-care chemoimmunotherapies and small molecules are initially efficacious but the majority of patients inevitably relapse with progressive disease (2). The only reliably curative therapy is allogeneic hematopoietic stem cell transplantation, but this comes with its own challenges, namely high morbidity and mortality, especially with a mainly elderly population and the difficulties associated with finding HLA-matched donors. CD19-directed cell therapies have shown promise, but with the exception of recent combination therapy trials where complete response rates can reach 40+% (3–5), only around one-fourth of patients with CLL treated with CD19-directed CAR T-cell therapy will achieve a complete remission (6–10). This was unexpected as the same therapy in pediatric acute lymphoblastic leukemia (ALL) has a complete response rate >80% (11–18). Further study to understand both tumor and T-cell biology in CLL is therefore warranted.

CLL is phenotypically, clinically, and genetically a highly heterogeneous disease. Patients stratify into fast and slow-progressing groups based on tumor immunoglobulin heavy chain variable region mutation status (19), driver and second-hit mutation status (20–23), and expression of ZAP70 (24–26) and CD38 (27–30). In addition, patients with CLL suffer from immune dysfunction mediated by maintenance of a protumor microenvironment (31–37) and endogenous T-cell dysfunctions including dysregulated cytokine production (38), reduced proliferation and cytotoxicity (39), and unstable immune synapse formation (40–42). The combined effects of CLL disease biology and impaired T-cell function influence the efficacy of autologous CAR T-cell therapies. Alterations in T-cell biology including terminal differentiation (43), exhaustion (39, 43–45), inverted CD4/CD8 ratios (43), impaired T-cell metabolic fitness (46, 47), and the advanced age of the patient population all impact clinical response rates (6, 48–50).

Significant work has been done to understand clinical progression in CLL. However, the field lacks a complete understanding of how tumor cells and engineered T cells interact and the subsequent impact on cell-based therapies. CLL cells residing in different biological niches have different phenotypes and activation profiles. The lymph node (LN), considered the “birth place” of CLL, is a highly organized, B-cell supportive environment (31–34), wherein CLL cells proliferate and maintain an activated profile (51–53) with higher expression of costimulatory and adhesion molecules (54–56). Peripheral blood (PB) CLL cells on the other hand, are generally unactivated and noncycling (50). Most CLL studies use PB-derived tumor cells, as these are the easiest to obtain. However, the differences in tumor phenotype between the LN and PB demonstrate that using only PB-derived CLL cells may inaccurately predict antitumor responses in other compartments.

CD40L-expressing, CD28ζ-signaling CAR T cells show enhanced CAR T-cell activation and improved the antigen-presenting cell (APC) phenotype of CLL cells (57). We therefore sought to use CD40L-mediated CLL activation to mimic the phenotype of LN-derived cells and directly test the impact of enhanced costimulation on 4-1BB–signaling CAR T-cell function. We address the gap in knowledge in understanding how CLL cells negatively impact allogeneic CAR T-cell products and how tumor cells from different compartments interact with these therapies. To study this, we developed an *in vitro* system wherein we show that CLL-mediated T-cell defects are also seen in a healthy donor–derived CAR T-cell setting; these dysfunctions are therefore not dependent on the endogenous T-cell defects seen in a patient setting, as detailed above. This is of interest as allogeneic therapies become more developed, particularly in a disease such as CLL where patients may benefit from a more-potent allogeneic CAR T-cell product. We show that these defects are rescuable, and attributable to poor costimulation by CLL cells, rather than permanent dysfunction or immune suppression. Furthermore, we show that LN-resident CLL cells are better targets for CAR T-cell lytic activity than circulating tumor cells, and this costimulatory phenotype can be modeled *in vitro*. Herein we describe the consequences of CLL/CAR T-cell interactions and show that the activated CLL compartment drives antitumor CAR T-cell responses. This represents a rational point of departure to design the next generation of cell-based therapeutics for this disease.

## Materials and Methods

### Antibodies and Flow Cytometry

The following antibodies and fluorescent reagents were used in this study: Live/Dead Fixable Aqua Dead Cell Stain Kit (Thermo Fisher Scientific*,* #L34957), Biotin-SP (long spacer) AffiniPure Goat Anti-Mouse IgG F(abʹ)_2_ Fragment Specific (Jackson ImmunoResearch, #115-065-072, RRID:AB_2338565), PE-streptavidin (BioLegend, #405204, RRID:AB_2921282), and PE-anti-CAR19 (Novartis, custom antibody). Additional antibodies can be found in Supplementary Table S1. Cell sorts were performed on a FACS Aria II machine managed by the University of Pennsylvania Flow Core.

### Cell Lines

The artificial APC (aAPC) cell lines were created and validated in-house by transducing K562 cells (ATCC, CCL-243, RRID: CVCL_0004) to stably express CD64, CD86, 4-1BBL, and either ROR1 or CD19. The CD40 ligand–expressing Ltk cell line (Ltk-CD40L; refs. 58, 59) was obtained from Dr. Cees van Kooten (Leiden University Medical Center, Leiden, the Netherlands). Cells were tested for *Mycoplasma* using the Cambrex MycoAlert kit (Promega, #LT07-118). The most recent test was in February, 2022. Furthermore, the cell lines have been regularly authenticated by the University of Arizona Genetics Core using short tandem repeat profiling.

### Paired LN and PB CLL Samples

Patients were enrolled in the Institutional Review Board–approved protocol: Natural History Study of monoclonal B-cell lymphocytosis, CLL/small lymphocytic lymphoma, lymphoplasmacytic lymphoma/Waldenstrom macroglobulinemia, and splenic marginal zone lymphoma (ClinicalTrials.gov number NCT00923507). Samples were obtained after written informed consent in accordance with the Declaration of Helsinki, and applicable federal regulations. PB mononuclear cells (PBMC) were isolated by density gradient centrifugation and cryopreserved. LN biopsies were mechanically disaggregated into single-cell suspensions and cryopreserved.

### CAR T-cell Generation

Normal donor apheresis products were obtained from the University of Pennsylvania Human Immunology Core. PBMCs were isolated via Ficoll density gradient centrifugation and cryopreserved in 70% OpTmizer 5 (OpT5) medium, 20% human AB serum, and 10% DMSO. T cells were isolated using the Miltenyi Pan T cell Isolation Kit (Miltenyi Biotec, #130-096-535) and cultured in OpT5 medium with T-cell expansion supplement (Thermo Fisher Scientific, #A1048501*)*, 2 mmol/L Glutamax, and 5% human AB serum supplemented with 100 U/mL hIL2 unless otherwise stated. Cells were activated using anti-CD3/anti-CD28 Dynabeads at a ratio of 3 beads:1 T cell. T cells were transduced with CAR-expressing lentivirus on day 1. Cells were counted and resuspended at 5.0 × 10^5^ cells/mL on days 3, 5, and 7. On day 9, cells were counted and cryopreserved. Absolute cell counts were obtained using the Luna fluorescence-based automatic cell counter (Logos Biosystems).

### Lentivirus Production

VSVg-pseudotyped anti-CD19 or anti-ROR1 CAR (CAR19 and CAR-ROR1, respectively) lentivirus was produced using HEK293T cells (ATCC, CRL-11268). Cells were seeded on day −1 in R10 medium (RPMI + 10% FBS + 1% Penicillin/Streptomycin). On day 0, the cells were transfected with VSVg, RSV/Rev, Gag/Pol, and CAR plasmids using lipofectamine 2000 (Thermo Fisher Scientific, #11668019). Supernatant was collected at 24 and 48 hours and concentrated using an ultracentrifuge overnight at 4°C and 8,861 Relative Centrifugational Force (RCF), followed by 2.5 hours at 4°C at 76,800 RCF. Virus was aliquoted and stored at −80°C.

### B-CLL Isolation

Primary CLL PBMCs were obtained from the Stem Cell and Xenograft Core at the University of Pennsylvania (Philadelphia, PA). B-CLL cells were isolated using the Miltenyi B-CLL Isolation Kit (Miltenyi Biotec, #130-103-466). A full list of CLL donors used can be found in Supplementary Table S2.

### B-CLL Cell Activation/Activated CLL Generation

Primary B-CLL cells were activated by plating them over confluent Ltk-CD40 L cells at 3.0 × 10^6^ cells/mL in Iscove's modified Dulbecco's medium (Thermo Fisher Scientific, #12440049*)* + 10% GemCell FBS (GeminiBio, #100-500). Cells were placed at 37°C for 6 hours before harvest.

### Cytotoxicity Assay

CAR T cells and donor-matched untransduced T cells were thawed and stained with 0.1 μmol/L carboxyfluorescein diacetate succinimidyl ester solution (Thermo Fisher Scientific, #C34554) according to manufacturer's instructions. Cells were counted and resuspended at 1.0 × 10^6^ cells/mL in OpT5 medium. B-CLL cells were isolated as described above and stained with 0.1 μmol/L Cell Trace Violet solution (Thermo Fisher Scientific*, #*C34557) according to manufacturer's instructions. CLL cells were counted, resuspended at 1.0 × 10^6^ cells/mL in OpT5 medium and cocultures were set up at the desired effector:target ratios. Untransduced cells were used as an alloreactivity control and aAPCs were used as a positive control. Assays using activated CLL cells or IL2 used CLL cells activated for 6 hours as described or had 100 U/mL IL2 added to the cocultures, respectively.

### Restimulation Assay

CAR T cells were sorted to obtain the live, CAR+ T-cell fraction and plated with aAPCs or primary CLL cells at a ratio of 3:1 APCs/CLL to CAR T cells at 1.0 × 10^6^ total cells/mL in OpT5 medium without cytokines. Stimulator cells were irradiated with 100 Gy prior to use. T cells were counted and stimulated on days 0, 5, and 10, and harvested on day 15.

### Trans-costimulation of CAR T Cells

CAR T-cell and CLL cocultures were set up as indicated in the restimulation assay. aAPCs expressing CD86 and 4-1BBL but an irrelevant CAR target antigen were added at a ratio of 3 aAPCs: 1 CAR T cell. Cocultures were left for 5 days and then assessed for CAR activation by flow cytometry.

### Intracellular Cytokine Analysis

CAR T cells were thawed, counted, and resuspended in OpT5 medium. CLL cells were isolated as described above. Cocultures were set up at a final concentration of 1.0 × 10^6^ total cells/mL in OpT5 medium with a 1:20 dilution of CD107a-FITC (BD Biosciences, #555800), 1 μg/mL brefeldin (BD Biosciences, #555029), and 2 μg/mL monensin (BD Biosciences, #554724) at a ratio of 3:1 Stimulator:CAR T cell. Cells were harvested after 6 or 12 hours and stained with Live/Dead Blue followed by surface staining for CAR, CD3, CD4, CD8, and CD19. Cells were fixed using the BD Intracellular Cytokine Staining Kit and Protocol (BD Biosciences, #554715) and stained intracellularly for IL2, TNFα, and IFNγ. Additional antibody information is given in Supplementary Table S1.

### Secreted Cytokine Detection

CAR T cells were cocultured with aAPCs or primary CLL cells for 24 hours. Supernatant was collected and stored at −80°C. Cytokine levels were assessed using the Luminex Human Th17 25-plex platform (Millipore) according to manufacturer's instructions.

### Statistical Analysis

Statistical analysis was performed using GraphPad Prism version 9 (GraphPad Software, San Diego, CA*).* All data were subjected to the D'Agostino-Pearson normality test. Nonparametric statistics were used where the sample size was too small or the data were non-normally distributed. Data were assessed for outliers using the ROUT method with a *Q* value of 1%. When outliers were identified they were removed from subsequent analysis. Statistical tests are indicated in the relevant figure legends along with exact *P* value. *P* values and adjusted *P* values as determined by the Holm-Sidak method to correct for multiple comparisons can be found in Supplementary Table S3. A *P* value <0.05 was considered statistically significant. Data are shown as mean ± SD.

### Data Availability Statement

The data generated during this study can be found within the article and Supplementary Data.

## Results

### CLL Cells Fail to Elicit Strong Effector Responses From Healthy Donor–derived CAR T Cells

**FIGURE 1:**
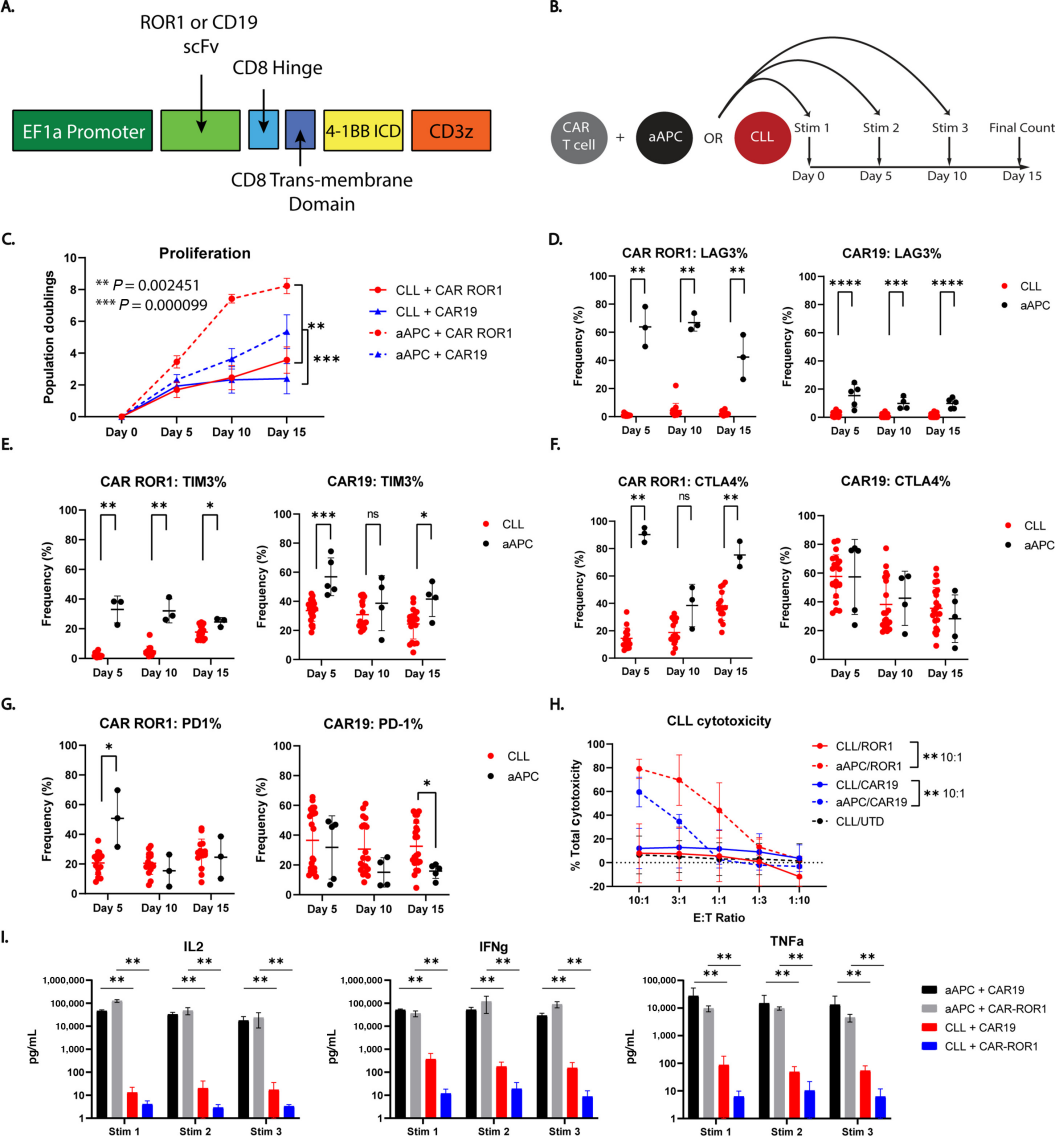
CAR-ROR1 and CAR19 T cells serially stimulated with CLL cells recapitulate clinically observed effector hypofunction. **A,** A schematic of the second-generation CAR construct used in these studies depicting the promoter, scFv, hinge, transmembrane, and intracellular signaling domains (ICD). **B,** An experimental schematic depicting the serial restimulation assay. CAR T cell indicates normal donor-derived CAR T cells, aAPC indicates the positive control cell line, and CLL indicates a primary tumor stimulus. **C,** Proliferation measured as population doublings of CAR-ROR1 and CAR19 T cells following restimulation with either aAPCs or CLL cells. The frequency of ROR1- or CD19-specific CAR T cells expressing the activation-associated molecules LAG-3 **(D)**, TIM-3 **(E)**, CTLA-4 **(F)**, and PD-1 **(G)** at each stimulation shows robust induction of expression with the aAPC but not CLL cells. CLL refers to CLL-stimulated cells while aAPC refers to CAR T cells stimulated with aAPCs. **H,** Cytotoxic function of CAR-ROR1 and CAR19 T cells against primary CLL cells or aAPCs after a 48-hour coincubation. UTD refers to the untransduced donor-matched control. **I,** Secreted cytokine production as measured by Luminex 24-hours after each stimulation. Concentration is given as pg/mL. Analyses were performed using the multiple Mann–Whitney test with Holm-Sidak correction. CAR-ROR1: *n* = 2 experiments, 5 CLL donors: (2655, 2761, 3416, 4487, 4625), CAR19: *n* = 5 experiments, 22 CLL donors: (1993, 3507, 3578, 3935, 3955, 4045, 4048, 4129, 4265, 4276, 4288, 4444, 5071, 5083, 5108, 5131, 5267, 5574, 5597, 5786, 5798, 5963). *P* values are as follows: **(D)** [ROR1: **, *P* = 0.002451; CAR19: ****, *P* = 0.000050 (left); ***, *P* = 0.000226; ****, *P* = 0.000025 (right)]. **E,** [ROR1: **, *P* = 0.002451; *, *P* = 0.036765; CAR19: ***, *P* = 0.000050; ns *P* = 0.365669; *, *P* = 0.021909]. **F,** [ROR1: **, *P* = 0.002451; ns *P* = 0.056373; CAR19: ns *P* = 0.746761 (left), 0.844946 (middle), 0.376935 (right)]. **G,** [ROR1: *, *P* = 0.004902; ns *P* = 0.352941 (middle); *P* = 0.654412 (right)]. **H,** [ROR1: **, *P* = 0.004396; CAR19: **, *P* = 0.008081]. **I,** IL2: [ROR1: **, *P* = 0.002451; CAR19: **, *P* = 0.004040 (left); *P* = 0.009524 (middle); *P* = 0.006061 (right)]. IFNγ: [ROR1: **, *P* = 0.002451; CAR19: **, *P* = 0.004040]. TNFα: [ROR1: **, *P* = 0.002451; CAR19: **, *P* = 0.004040 (left, middle), *P* = 0.006061 (right)]. (*, *P* < 0.05; **, *P* < 0.01; ***, *P* < 0.001; ****, *P* < 0.0001).

To study CLL/CAR T-cell interactions, second-generation CD19- and ROR1-directed CAR T cells with a 4-1BB intracellular signaling domain (Fig. 1A) were serially stimulated with primary CLL cells every 5 days for three total stimulations (Fig. 1B). A ROR1-directed CAR was selected since this molecule is under development for treatment of CLL and other malignancies (60, 61), and the CD19-directed CAR was chosen to validate the study results as it remains the gold standard for the field. An irradiated K562-based aAPC expressing the relevant CAR target antigen was used as a positive control. These aAPCs stimulate potent effector activity including proliferation and cytokine production (62–64). Because the aAPCs are transduced to express the CAR target antigen, expression of these proteins may differ from endogenous expression levels (Supplementary Fig. S1A). The expression of CD19 is comparable between the aAPCs and the endogenous expression on the CLL cells. However, the expression level of ROR1 on the aAPCs is higher than on the CLL tumor. However, variability in expression of both proteins is seen on the tumor cells, with more variable expression of ROR1 than CD19. The aAPCs also express CD86 and 4-1BBL in addition to either ROR1 or CD19. However, CAR19 T cells proliferate similarly in response to both aAPCs with CD86/4-1BBL and aAPCs that only express CD19 (Supplementary Fig. S1B). Furthermore, we chose to use a 4-1BB–signaling CAR due to the clinical success of Kymriah, a CD19-directed, 4-1BB–signaling CAR, and the observation that a CD28-signaling CD19-directed CAR also demonstrated similarly reduced proliferation in response to CLL cells (Supplementary Fig. S1C).

Following three consecutive CLL stimulations, we found that both CD19- and ROR1-directed CAR T cells proliferated significantly less than aAPC-stimulated cells (Fig. 1C). Furthermore, both CAR T cells showed delayed, low-level upregulation of the inhibitory receptors LAG3 (Fig. 1D) and TIM3 (Fig. 1E) while these proteins were strongly induced following aAPC stimulus. CAR-specific differences were seen in expression patterns of CTLA4 and PD-1 (Fig. 1F and G), but the overall reduction in activated phenotype was consistent. Cytotoxic function after a 48-hour coculture was also impaired in CLL-stimulated CAR T cells but remained intact in aAPC stimulated cells (Fig. 1H). Finally, aAPC-stimulated CAR T cells produced higher levels of cytokines than the matched CLL-stimulated cells (Fig. 1I). Together, these data confirmed that we can model CLL-induced T-cell nonresponsiveness *in vitro* using primary CLL cells and healthy donor–derived CAR T cells, and that this phenotype is inducible irrespective of CAR target antigen. Furthermore, this confirmed that CLL cells induce hypofunction in a CAR T-cell setting, not just in autologous CLL-derived T cells. Having determined that these functional defects were CAR independent, we chose to focus our efforts on the relatively understudied ROR1-directed CAR, validating major findings with CAR19 as needed.

### CLL-induced CAR T-cell Dysfunction is Due to Insufficient Activation

**FIGURE 2:**
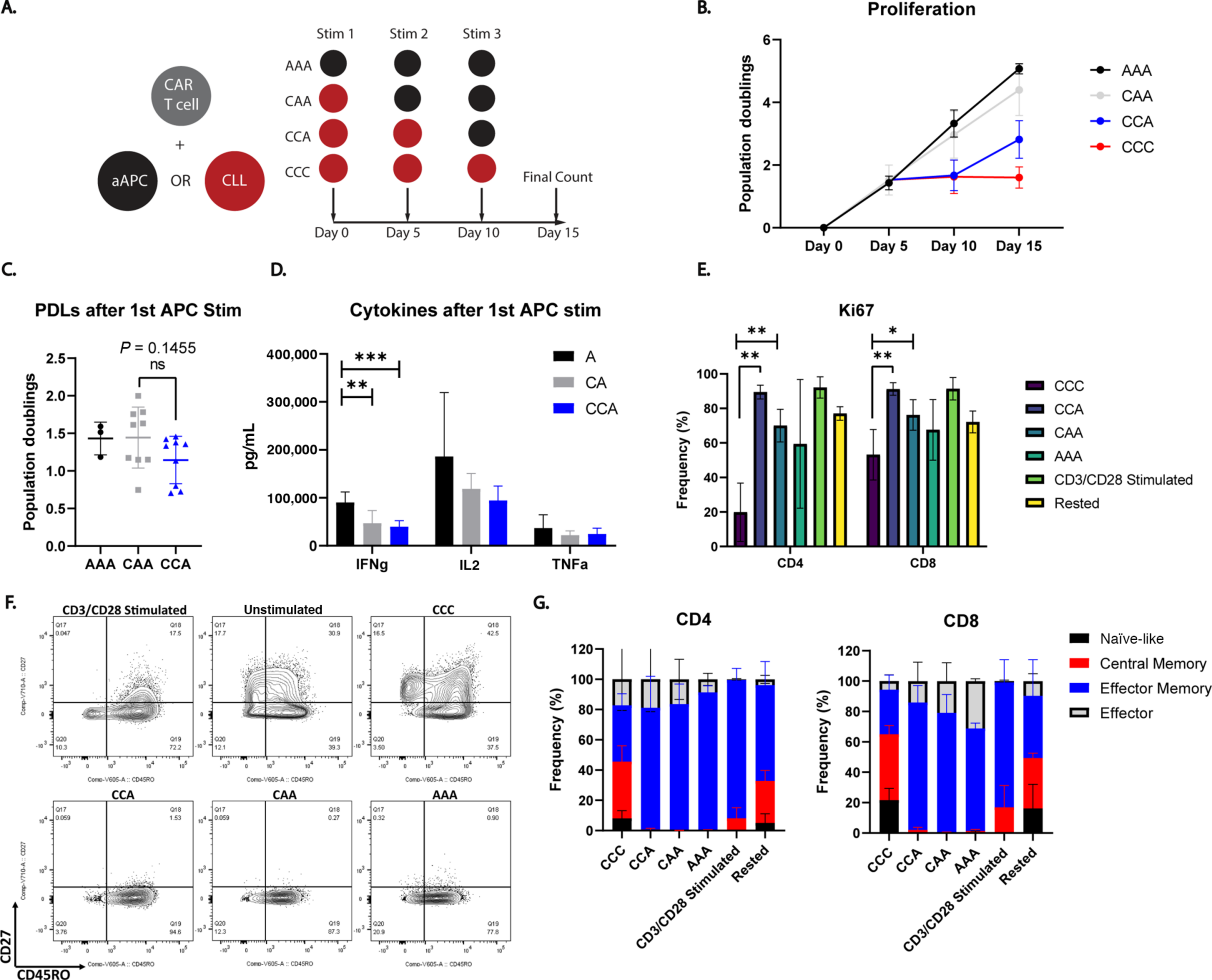
CLL-mediated CAR-ROR1 dysfunction is due to insufficient activation as opposed to permanent defects. **A,** An experimental schematic depicting the APC switching assay. Red and black circles indicate whether CAR T cells were challenged with CLL cells or aAPCs, respectively for a total of three times. *AAA:* Three successive aAPC stimulations. *CAA:* One CLL stimulation followed by two successive aAPC stimulations. *CCA:* Two CLL stimulations followed by one aAPC stimulation. *CCC:* Three successive CLL stimulations. **B,** Proliferation of CAR-ROR1 T cells as measured on days 5, 10, and 15 of the APC switching restimulation assay. Proliferation is given as population doublings. **C,** The number of population doublings each group underwent after receiving one aAPC stimulus. For the AAA condition, this was day 5, for CAA this was day 10, and for CCA this was day 15. No significant difference is observed between the groups, indicating no sustained defect after successive CLL interactions. **D,** The levels of IFNg, TNFa, and IL2 produced by each group after receiving one aAPC stimulus as described in **C**. **, *P* = 0.013225; ***, *P* = 0.000535. **E,** The frequency of Ki67^+^ CAR-ROR1 T cells amongst the different restimulation conditions. CD3/CD28 stimulated indicates CAR T cells stimulated with anti-CD3/CD28 beads as a positive control and “Rested” indicates CAR T cells that were thawed and rested overnight as an unactivated cell control. **F,** Representative flow cytometry plots depicting the differentiation state of AAA, CAA, CCA, CCC, unstimulated CAR-ROR1 T cells, and CD3/CD28 bead-activated CAR-ROR1 T cells as determined by CD27 and CD45RO staining. **G,** Quantification of the frequencies of each differentiation state defined as follows: Effector: CD27^−^CD45RO^−^. Effector Memory: CD27^−^CD45RO^+^. Central Memory: CD27^+^CD45RO^+^. Naïve-like: CD27^+^CD45RO^−^. CD4^+^ CAR-ROR1 T cells are depicted on the left and CD8^+^ CAR-ROR1 T cells are depicted on the right. **C** was analyzed using an unpaired, two-tailed *t* test. **D** and **E** were analyzed using multiple Mann–Whitney test with Holm-Sidak correction. *n* = 3 experiments, 5 CLL donors: (3578, 3955, 4288, 4444, 4279). (*, *P* < 0.05; **, *P* < 0.01).

We next sought to determine the causes of this hypofunction. On the basis of the body of work detailing T-cell exhaustion in CLL, we hypothesized that CLL-mediated dysfunction was a sustained, defective state, stabilized by successive interactions with CLL cells. We tested this by stimulating CAR-ROR1 T cells with CLL cells once or multiple times and then switched to an aAPC stimulus (Fig. 2A). Sustained hyporesponsiveness of CAR T cells against strongly stimulatory targets following one or more encounters with CLL cells would be highly suggestive of permanent dysfunction. However, no differences in the magnitude of the proliferative response were observed after switching to an aAPC stimulus in any of the stimulation conditions (Fig. 2B and C). Switching to an aAPC also restored the breadth of the secreted cytokine repertoire, although a slight decrease in cytokine level was observed following repeated CLL stimulations (Fig. 2D). In addition, we observed a near absence of cells entering the cell cycle as denoted by Ki-67 positivity, a phenotype which was rescued by aAPC stimulation (Fig. 2E). We then used flow cytometry to assess the differentiation state of the CAR T cells at the end of the coculture. While all aAPC-stimulated CAR T cells underwent memory or effector differentiation, CLL cells failed to induce progressive T-cell differentiation (Fig. 2F and G). Together, these data indicated that CLL cells insufficiently activate even second-generation CAR T cells, explaining the observed lack of effector responses. This suggested that CLL cells either lack a positive regulator of CAR T-cell responses which was then provided by an aAPC, or that CLL cells negatively regulate CAR T cells which can be rescued by removing the T cells from a CLL-dense environment. The ability to rescue CAR T-cell function indicated that we could improve anti-CLL responses if we enhanced T-cell activation.

### Strong Antigenic Stimulation is Sufficient to Overcome CAR T-cell Activation Defects

**FIGURE 3:**
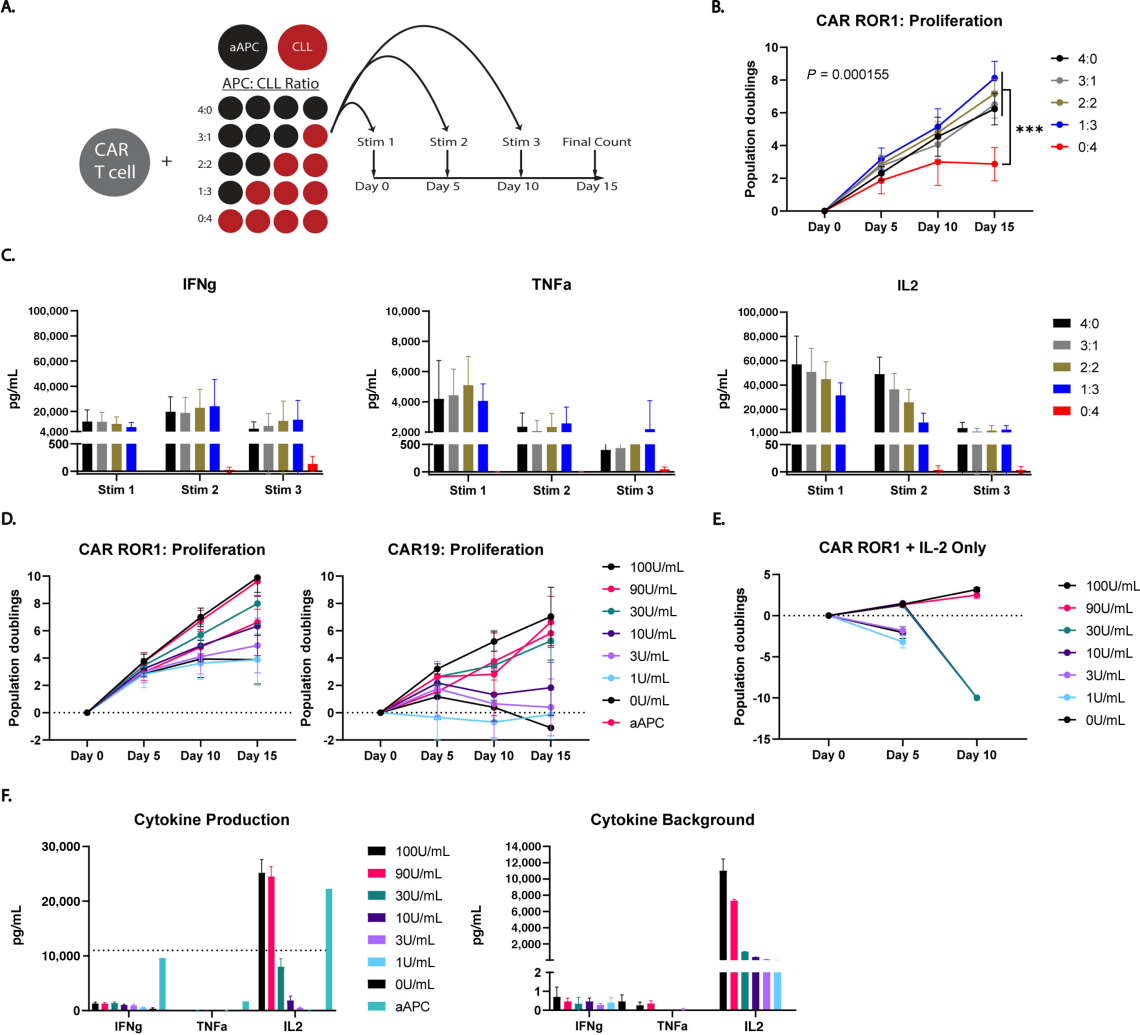
Strong antigenic stimulation partially rescues CLL-mediated insufficient activation. **A,** An experimental schematic depicting the mixed APC assay. aAPCs and CLL cells were mixed together and CAR-ROR1 T cells were stimulated with the indicated ratios of aAPC to CLL cells. 4:0 and 0:4 indicate that CAR T cells were stimulated with aAPC or CLL cells only, respectively. 3:1 indicates a mix of 75% aAPCs and 25% CLL cells and 2:2 indicates a 50/50 mix of the two cell types CLL cells do not inhibit the proliferative **(B)** or cytokine secretion response **(C)** of CAR T cells to aAPC. **D,** Mid-to-high dose IL2 rescues the proliferative response of CAR T cells against CLL cells. Proliferation of CAR-ROR1 T cells (left) and CAR19 T cells (right) is given as population doublings during the restimulation assay with the given quantity of IL2 supplemented into the culture medium. **E,** Proliferation of CAR-ROR1 T cells incubated with the given quantity of IL2 alone in the absence of antigen. Proliferation is given as population doublings. **F,** Cytokine production of CAR-ROR1 T cells on day 15 of the restimulation assay with the indicated amount of supplemented IL2 (left). Background levels of IFNg, TNFa, and IL2 present in the media with exogenous IL2 added (right). The dotted line on the left graph indicates the background IL2 levels for the 100 U/mL condition with no CAR T cells present. **B** was analyzed using the multiple Wilcoxon matched-pairs signed rank test with Holm-Sidak correction. **B**: *n* = 3 experiments, 4 CLL donors: (4265, 4516, 4567, 5574). **C**: *n* = 5 CAR T-cell donors. **D** (left): *n* = 3 experiments, 3 CLL donors: (4526, 4567, 5574). **D** (right): *n* = 1 experiment, 1 CLL donor: (4625). **E**: *n* = 1 experiments, 4 CLL donors: (4265, 4516, 4567, 5574). **F**: *n* = 3 CAR T-cell donors (***, *P* < 0.001).

We next hypothesized that impaired CAR T-cell activation was due to active immunosuppression. To test this, we developed a mixed coculture assay wherein CAR T cells were stimulated with aAPCs and CLL cells mixed at graded ratios (Fig. 3A). All cultures containing any proportion of aAPCs proliferated (Fig. 3B) and produced cytokines at similar levels (Fig. 3C) regardless of the frequency of CLL cells in the culture (Fig. 3B). This suggested that CLL cells do not prevent CAR T-cell activation in the presence of a potent stimulus.

### Exogenous IL2 Supplementation Overcomes Impaired CAR T-cell Proliferation in CLL Cocultures

Although proliferation was unaffected by CLL cells in the mixed culture assay (Fig. 3B), we observed a trend wherein IL2 production decreased with increasing numbers of CLL cells (Fig. 3C, right). We hypothesized that this limited IL2 secretion may drive insufficient T-cell activation. To determine whether IL2 was limiting, we performed a restimulation assay while supplementing graded doses of IL2 to the cultures. We found that this restored both CAR-ROR1 and CAR19 proliferation in response to CLL cells in a dose-dependent manner (Fig. 3D). This response was at least partially antigen-driven as IL2 supplementation alone did not result in the same levels of T-cell proliferation (Fig. 3E). Finally, we assessed cytokine production in the IL2 supplemented cultures and observed partial restoration of the secreted cytokine repertoire (Fig. 3F).

### IL2 Enhances the Stimulatory Phenotype of CLL Cells

To discern a mechanism for the improved CAR T-cell responses against CLL cells with IL2 addition, we immunophenotyped primary CLL cells incubated for 5 days with high-dose (100 U/mL) IL2 (Supplementary Fig. S2A). We found that the IL2R alpha-chain (CD25) was expressed on >80% of the tumor cells in 18 of 20 CLL donors (Supplementary Fig. S2B). This suggested that these CLL cells may have a functional, signaling IL2R; this could lead to phenotypic changes in response to IL2. We found that while IL2 stimulation did not alter the expression of inhibitory ligands PD-L1 and PD-L2 (Supplementary Fig. S2C), expression of the costimulatory molecules CD80 and CD86 was significantly augmented (Supplementary Fig. S2D). The expression of the adhesion molecules CD54 and CD58 was similarly increased (Supplementary Fig. S2E). Furthermore, we observed induction of CLL cell proliferation by IL2 (Supplementary Fig. S2F). Together, these data show that CLL cells acquire a phenotype consistent with B-cell activation when incubated with IL2. We postulated that this prostimulatory phenotype could explain the improved CAR T-cell responses observed in Fig. 3.

### Additional Costimulation Enhances Second-generation CAR T-cell Activation

Given that IL2 significantly boosted the expression of costimulatory molecules on CLL cells, we sought to determine whether enhanced costimulatory receptor signaling could facilitate better activation of second-generation CAR T cells. To test this, we provided stimulation via CD86 and 4-1BBL using a bystander aAPC that did not express the CAR target antigen (Supplementary Fig. S3A). We referred to this system as trans-costimulation to differentiate from costimulation provided by the target cell itself (costimulation *in cis*). Trans-costimulation enhanced CAR-ROR1 T-cell proliferation (Supplementary Fig. S3B) and the activation phenotype of both CAR-ROR1 and CAR19 T cells as assessed by PD-1, TIM-3, and CTLA-4 upregulation in CLL cocultures (Supplementary Fig. S3C–S3E). Furthermore, a shift toward a central memory immunophenotype indicated rescued CAR T-cell differentiation (Supplementary Fig. S3F and S3FG). Together, these data show that additional costimulation positively impacts second-generation CAR T cells and implicates an improved APC phenotype in driving anti-CLL T-cell responses.

### LN-derived CLL Cells Have an Improved Stimulatory Phenotype Compared with PB-derived CLLs

**FIGURE 4:**
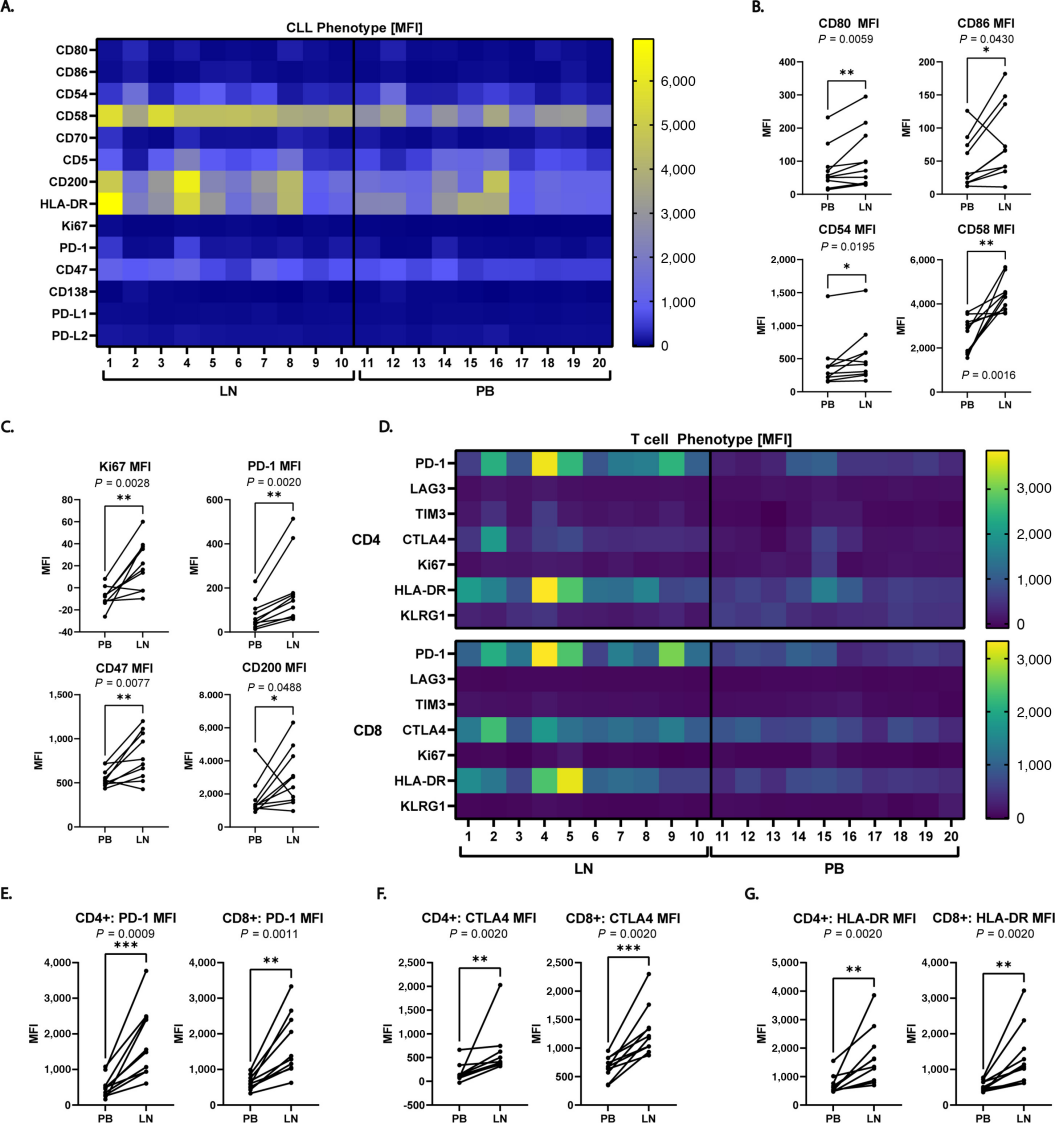
The CLL LN provides a pro-T-cell stimulatory environment. **A,** Heatmap showing the phenotype of 10 matched LN- and PB-derived CLL cells given as mean fluorescence intensity (MFI). **B,** APC phenotype given as comparisons of expression of costimulatory molecules CD80, CD86, CD54, and CD58 between PB- and LN-derived CLL cells. Expression is given as MFI. **C,** Comparison of B-CLL activation molecules Ki67, PD-1, CD47, and CD200 on PB compared with LN-derived CLL cells. Expression is given as MFI. **D,** Heatmap depicting the T-cell activation phenotype of CD4 T cells (top) and CD8 T cells (bottom) derived from the LN compared with the PB. **E,** Expression of PD-1 on CD4^+^ (left) and CD8^+^ (right) endogenous T cells from the LN and PB from the 10 matched samples in **A**. Expression is given as MFI. **F,** Expression of CTLA-4 on CD4^+^ (left) and CD8^+^ (right) endogenous T cells from the LN and PB from the 10 matched samples in **A**. Expression is given as MFI. **G,** Expression of HLA-DR on CD4^+^ (left) and CD8^+^ (right) endogenous T cells from the LN and PB from the 10 matched samples in **A**. Expression is given as MFI. **B** (top left and bottom left), **C** (top right and bottom right), **F**, and **G** were analyzed using the two-tailed Wilcoxon matched-pairs signed rank test. The remaining analyses in **B** and **C** and **E** were analyzed using a two-tailed paired *t* test. (*n* = 10 LN/PB pairs, *, *P* < 0.05; **, *P* < 0.01; ***, *P* < 0.001).

It is well established that a significant fraction of LN-resident CLL cells expresses the cell-cycle marker Ki67, while PB CLL cells are largely quiescent (50, 51, 53, 54, 56). We hypothesized that LN-resident CLL cells also express higher levels of the adhesion and costimulatory molecules these second-generation CAR T cells still rely on. To assess this, we examined 10 paired LN and PB samples from patients with treatment-naïve CLL (Table 1) for expression of costimulatory and inhibitory ligands on tumor cells and their matching receptors on T cells. As expected, LN-derived CLL cells had a more activated, stimulatory phenotype than the matched PB CLL cells (Fig. 4A). The LN-derived tumor cells expressed higher levels of costimulatory and adhesion molecules (Fig. 4B) as well as molecules associated with B-cell activation, including Ki67, PD-1, CD200, and CD47 (Fig. 4C). In addition, both CD4^+^ and CD8^+^ T cells from the LN were more activated than the matching PB T cells (Fig. 4D). Both CD4^+^ and CD8^+^ T cells taken from the CLL LN had higher expression of PD-1 (Fig. 4E), CTLA-4 (Fig. 4F), and HLA-DR (Fig. 4G) than those from the PB. Coexpression of these molecules is highly suggestive of an activated T-cell phenotype. We therefore hypothesized that in contrast to quiescent PB CLL cells, strong stimulation provided by this LN-resident CLL population drives anti-tumor CAR T-cell responses in patients.

**TABLE 1:** Paired LN/PB patient samples. List of the 10 paired samples for which we have both PB- and LN-derived CLL cells. Age, sex, Rai Stage, IGHV mutational status, and cytogenetic abnormalities for each donor are indicated

			Rai	IGHV	
Sample ID	Sex	Age	stage	status	FISH
1	F	55	1	U	13q
2	M	43	2	U	17p
3	M	48	3	U	17p
4	M	48	4	U	13q
5	M	50	3	U	11q
6	F	49	1	M	13q
7	F	57	3	U	13q
8	M	73	4	M	11q
9	F	55	3	U	t12
10	M	47	4	M	17p

### LN-derived CLL Cells Elicit Stronger CAR T-cell Effector Responses Than PB-derived CLLs

**FIGURE 5:**
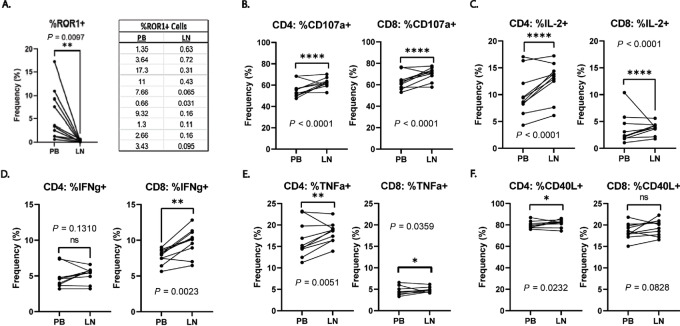
LN-derived CLL cells induce stronger CAR T-cell degranulation and cytokine production than PB-derived CLLs. **A,** The frequency of ROR1+ tumor cells in the LN and PB compartments. Raw numbers are given on the right*.***B,** Degranulation of CD4^+^ and CD8^+^ CAR19 T cells as measured by frequency of CD107a following a 6-hour stimulus with paired LN- or PB-derived CLL cells as described in Table 1. **C–E,** Frequency of IL2+, IFNγ+, and TNFα+ CAR19 T cells following a 6-hour stimulus with the LN and PB CLL pairs. **F,** CAR T-cell activation as measured by frequency of CD40L^+^ cells. Each data point represents an average of four CAR T-cell donors stimulated with each LN/PB CLL pair over the course of two experiments. **B** (left), **C** (right), **D** (right), **E**, and **F** (left) were analyzed using a two-tailed Wilcoxon matched-pairs signed rank test. **A**, **B** (right), **C** (left), **D** (left), and **F** (right) were analyzed using a two-tailed paired *t* test. (*n* = 10 LN/PB pairs, 4 CAR T-cell donors, two experiments. *, *P* < 0.05; **, *P* < 0.01; ****, *P* < 0.0001).

Next, we sought to directly test whether CLL cells from the LN stimulated improved CAR T-cell responses compared with those from the PB. We stimulated CAR19 T cells for 6 hours with the matched LN/PB CLL pairs described in Table 1. The ROR1-directed CAR was not used because none of the 10 donors expressed the ROR1 antigen in the LN compartment. While most samples had ROR1+ cells, this population was entirely restricted to the PB tumor cells (Fig. 5A). We assessed cytokine production and degranulation as a proxy to cytolytic activity using flow cytometry. We observed an increase in degranulation of both CD4 and CD8 CAR19 T cells following a LN CLL stimulation compared with a PB CLL stimulation (Fig. 5B). Furthermore, LN-derived CLL cells elicited more IL2, IFNg, and TNFa production than their paired PB-derived counterparts (Fig. 5C–E). CD4^+^ CAR19 T cells also showed strong activation responses as indicated by CD40 L upregulation (Fig. 5F). Together, these data show that the enhanced costimulatory phenotype of CLL tumor in the LN corresponds to improved CAR T-cell activation and effector responses, further highlighting the CLL costimulatory phenotype as having a major impact on CAR T-cell activation.

### A Strong CLL Costimulatory Phenotype can be Induced *In Vitro*

**FIGURE 6:**
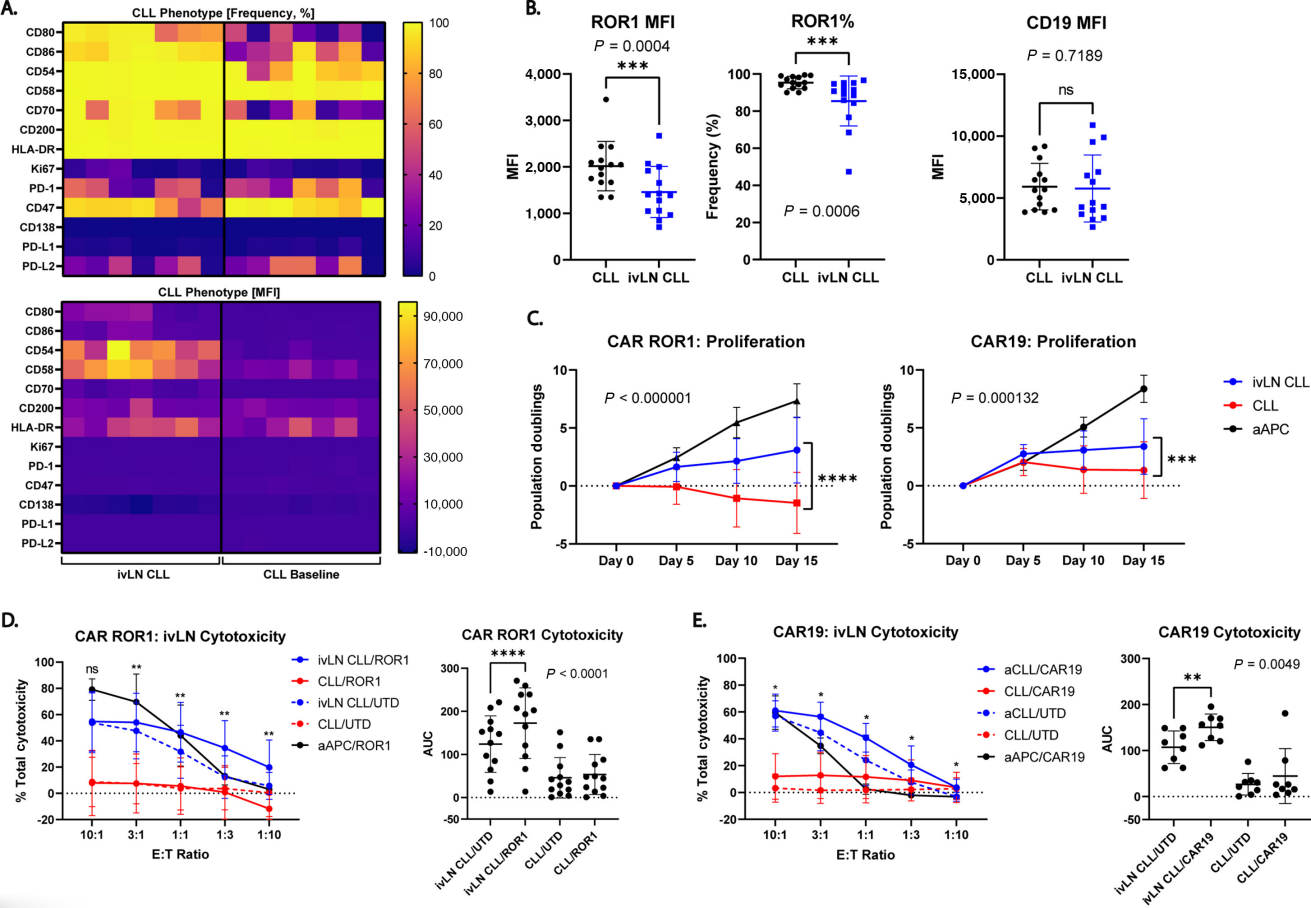
Strongly costimulatory CLL cells stimulate robust CAR T-cell effector responses. **A,** Heatmaps showing the phenotype of aCLLs compared with resting CLL cells, looking at both frequency (top) and MFI (bottom) of the markers indicated. **B,** Changes in expression level of ROR1 (left, middle) and CD19 on resting and aCLL cells. **C,** Proliferation of CAR-ROR1 (left) and CAR19 (right) T cells following serial stimulations with resting CLLs, a, or aAPCs. Proliferation is given as population doublings. **D,** Cytotoxicity of CAR-ROR1 and untransduced donor-matched control (UTD) T cells after a 2-day stimulation with either aCLLs or resting CLLs. Asterisks indicate comparisons between aCLL/CAR-ROR1 versus aCLL/UTD (left). Comparison of AUC for each condition from the data on the left. **E,** Cytotoxicity of CAR19 and UTD T cells after a 2-day stimulation with either aCLLs or resting CLLs. Asterisks indicate comparisons between aCLL/CAR19 versus aCLL/UTD (left). Comparison of AUC for each condition from the data on the left. **B**, **C** (right), **D** (left), and **E** (left) were analyzed using the Wilcoxon matched-pairs signed rank test with Holm-Sidak correction. **C** (right), **D** (right), and **E** (right) were analyzed using a paired two-tailed *t* test. [**B** (left): *n* = 4 experiments, 6 CLL donors: (2655, 4167, 4516, 4556, 4625, 4794), **B** (right): *n* = 2 experiments, 5 CLL donors: (2655, 4405, 4556, 4625, 6342), **C**: *n* = 2 experiments, 7 CLL donors: (2655, 2656, 4121, 4405, 4567, 4625, 4684), **D**, **E**: *n* = 2 experiments/CAR, 8 CLL donors: (2655, 2761, 2771, 3416, 4405, 4419, 4556 4625), *, *P* < 0.05; **, *P* < 0.01; ***, *P* < 0.001; ****, *P* < 0.0001].

Because donation of LN-derived CLL cells is an invasive procedure, we sought to model the LN phenotype *in vitro*. To achieve this, we tested whether CD40L-mediated activation would approximate the LN-resident CLL phenotype by stimulating primary CLL cells for only 6 hours with a human CD40 ligand–expressing epithelial cell line. Our results revealed that a brief 6-hour stimulation resulted in upregulation of costimulatory, adhesion, and activation-associated molecules in CLL cells, in line both with prior CD40L activation studies (51, 56, 65) and the patient LN CLL phenotype (Fig. 6A). CD40L-activated CLL cells also maintained expression of both ROR1 and CD19 antigens, although we observed a slight decrease in ROR1 expression (Fig. 6B). However, based on cell phenotype, we determined that CD40L-activated CLL cells (aCLL) mimic the LN-resident CLL cell costimulatory phenotype and can be used to study the impact of improved costimulation on CAR T-cell effector responses.

### Activated CLL Cells Drive Potent Antitumor CAR T-cell Responses

To test our hypothesis that enhanced costimulation on LN-derived CLL cells directly mediates CAR T-cell effector function, we performed repeat stimulation assays using aCLL cells and found that both CAR-ROR1 and CAR19 T cells had enhanced proliferation with an aCLL stimulus compared to unactivated CLL cells (Fig. 6C). We further assessed the ability of CAR T cells to degranulate (Supplementary Fig. S4A) and produce cytokines (Supplementary Fig. S4B–S4D). CAR19 CD8^+^ T cells displayed increased degranulation after a 6-hour aCLL stimulus indicating enhanced cytotoxic function while CAR ROR1 T cells had a mixed phenotype (Supplementary Fig. S4A). We also observed inconsistent expression of IL2, IFNγ, or TNFα between the CARs at the 6-hour timepoint (Supplementary Fig. S4B–S4D). We next determined whether aCLL cells were susceptible to CAR-mediated lysis. Here we confirmed that that both CAR-ROR1 (Fig. 6D) and CAR19 T cells (Fig. 6E) preferentially lysed aCLL cells over the matched unactivated CLL cells. Furthermore, we observed enhanced lysis at lower effector:target ratios with aCLL stimulation. Our results demonstrate that CAR T cells effector responses are directly affected by costimulatory molecule expression on the CLL tumor. Furthermore, these data suggest that CAR T cells may preferentially target activated CLL cells, such as those found in the LN. Thus, the clinical efficacy of CART19 therapy in CLL may be explained by the lysis of LN-resident CLL cells as PB-derived CLL cells are poor stimulators of CAR T-cell activity.

## Discussion

Despite the relative success of CD19-directed CAR T-cell therapy in treating B-cell malignancies, only a minority of patients with CAR T cell–treated CLL will be classed as complete responders, highlighting a remaining unmet need for curative therapies. Although there exists a body of work on how CLL cells impact endogenous T-cell function, little work has been done to understand how primary CLL cells affect cell-based therapies. With the advent of transgenic-TCR (T-cell receptor) and CAR-modified T cells, as well as the rising popularity of allogeneic or “off-the-shelf” cell therapies, there remains a need to understand how CLL cells impact healthy donor–derived T cells. Furthermore, understanding the differences between the immune compartments in which CLL cells reside and how each impacts the efficacy of cell-based therapies is critically important. This study aimed to understand how primary CLL cells negatively impact allogeneic CAR T-cell products and, subsequently, identify how the tumor microenvironment (TME) drives antitumor responses in a patient population. This work will allow the field to enhance existing therapies to capitalize on TME modulation, improve CAR T-cell therapeutic responses, and ultimately provide more successful treatments for patients with CLL.

It is well documented that CLL cells negatively impact both endogenous and allogeneic T cells, regardless of TCR specificity. Herein, we demonstrate that even second-generation CAR T cells exhibit dysfunctions against primary tumor cells. However, these dysfunctions are not permanent; rather they are the result of insufficient CAR T-cell activation. While immunosuppression mediated by CLL cells may abrogate CAR T-cell function, in the presence of strong, positive activation signals such as stimulation with an aAPC or addition of high-dose IL2, CAR T-cell effector functions remain intact. This improvement is mediated by enhanced APC phenotypes as defined by increased expression of costimulatory and adhesive molecules. We show that this APC phenotype is maintained by LN-resident CLL cells and is lacking in the PB-derived CLL population. Furthermore, this phenotype directly impacts the activation state of T cells derived from these compartments. By stimulating CAR T cells with aCLL cells, we enhanced anti-CLL effector functions, including proliferation and cytotoxicity, both of which are predictive of better clinical response. These findings further highlight an apparent paradox—that 26% of patients with CLL will have a durable complete response to CAR T-cell therapy. Our data suggest that in this setting, CAR T cell–mediated remissions are largely driven by a LN response, initially eliminating the proliferative progenitor CLL cells and subsequently becoming highly activated, allowing for peripheral tumor clearance.

Autologous CLL T cells are well documented to have impaired proliferative and effector responses, dysregulation of the CD4/CD8 immune balance, and a terminally differentiated phenotype (39, 40, 66–68). While most studies and clinical trials have used autologous T cells, the results are mixed, likely due to disease heterogeneity between patients. By using normal donor-derived CAR T cells, we are able to focus on tumor-mediated mechanisms without the confounding variable of autologous T-cell function. Furthermore, this work is directly applicable to allogeneic cell therapy, which is a rapidly growing field of study. Understanding how CLL tumor negatively impacts even healthy T-cell activation will further enable these allogeneic cell therapies.

The heterogeneity of primary CLL tumors has made it challenging to engineer representative murine or cell-based models. While murine systems to study CLL do exist, they are complicated and time consuming to develop (69, 70). Another difficulty in studying primary CLL is that the cells do not grow autonomously *in vitro* without concomitant CD40 ligation and cytokine/CpG addition (58, 71–74) or Epstein-Barr Virus (EBV) transformation, such as with the OSU-CLL cell line (75). Our study addressed this gap by focusing on primary CLL cells as a biologically and translationally relevant tool to study this disease. Using our primary tumor cell bank, we consistently observed that CLL cells prevent CAR T-cell activation and that the poor stimulatory phenotype of CLL cells explains this impairment. The consistency between CLL donors and CAR19 and CAR-ROR1 cells validates our conclusions, suggesting a conserved activation defect despite sample heterogeneity. Further our finding that the LN compartment drives CAR T-cell responses highlights a need for the CLL field to better understand the TME in this disease.

It is of note that, clinically, large LNs are cleared more slowly than the PB or bone marrow. This begs the question, if the LN is in fact the site of active T-cell killing is it also a sanctuary site for the tumor? Although we can model a LN-like CLL phenotype *in vitro*, we cannot model the entire associated TME. Thus, this *in vitro* system lacks cells that may provide support to the tumor or negatively regulate CAR T-cell function, via either secreted or contact-dependent mechanisms. It is logical to assume that the LN, traditionally a home for mature B cells, provides prosurvival signals to the tumor. Furthermore, being a tumor-supportive environment, it is likely that the *in vivo* LN has T-cell immune-suppressive qualities. More complete models of the tumor microenvironment both *in vivo* and *in vitro* are required to systematically address the relative contributions of cell types and secreted factors from this tissue.

The data presented in our study bring to mind prior work wherein vaccination of patients with CD40L-expressing CLL cells enhanced tumor killing by endogenous T cells (76, 77). However, the effects of these treatments were time limited, and ceased to be effective once the CD40L-expressing tumor was no longer present. These data suggest that priming of CD4^+^ T cells by CD40 L likely drove this response, but once the activating signal was removed, T cells again became unresponsive. In our study, we preactivated CLL cells with CD40 L before removing the source of CD40L, such that the CAR T cells were impacted by the tumor rather than the CD40L-expressing cell line. However, the sensitization of CLL cells to T-cell killing in the prior studies is in accordance with our own findings, wherein CAR T cells better proliferate and kill CD40L-activated CLL cells (see Fig. 6C–E). Interestingly, we also see bystander activation of untransduced T cells as measured by cytotoxicity in response to the activated CLL stimulus. This supports the findings of the above studies, wherein not only are T cells activated allowing for cytotoxic function, but the activated CLL cells themselves serve as better targets for T-cell killing. This highlights the requirement of costimulatory signaling for activation of TCR-directed T cells. The CAR T-cell field then capitalized on such findings to create second-generation CARs that contain costimulatory domains, enabling killing of target cells even in the absence of costimulatory molecules on the cell surface—as is the case in CLL. The low costimulatory ligand expression on CLL tumor greatly limits native T-cell killing capacity, and, as our data indicate, can impact CAR T-cell function, since enhancing expression of costimulatory ligands rescues CAR T-cell responses.

Surprisingly, this study identifies a need for additional costimulatory signaling even in the context of a second-generation CAR that already contains a 4-1BB costimulatory signaling domain. This is highlighted by the improved activation and killing observed with aCLL-stimulated CAR T cells as well as the improved activation phenotype of CAR T cells in a trans-costimulation setting. The introduction of costimulatory molecules on CAR T cells to allow for cross-stimulation of CAR T-cell products will both enhance CAR T-cell function and stimulate the endogenous immune system (78). This finding also calls into question whether CAR design has been fully optimized to provide adequate costimulatory signaling. Studies have shown that the addition of the costimulatory molecules CD80 or 4-1BBL to CAR19-BBζ or CAR19-28z T cells, respectively, enhanced CAR T-cell persistence and tumor clearance in a mouse model of ALL (79). The addition of 4-1BBL extended persistence of the 28z CAR compared with the 28z CAR alone, and also maintained higher numbers of CD8^+^ CAR T cells long term. Both the CD80-expressing and 4-1BBL–expressing CARs demonstrated marked reduction in exhaustion. These findings, while shown in an ALL model, demonstrate that improving the type and level of costimulation has positive impacts on second-generation CAR T-cell function, as measured by T-cell killing, persistence, and overall antitumor activity. This study confirms the importance of optimal costimulation, even in a setting where CAR T-cell function is “optimized” and yields impressive clinical responses. Extrapolating out to a CLL setting where responses to CAR T-cell therapy are lacking, our data support the investigation of adding costimulatory molecules to CAR lentiviral construct design as a method to improve response rates. CD40 L has also been added to CAR19 T cells and demonstrated improved antitumor efficacy via endogenous immune system activation in murine models of leukemia and/or lymphoma (78). In a CLL setting, this could also function as an indirect mechanism to enhance the native immune response and may activate CLL cells to upregulate costimulatory molecules *in vivo.* Combining cellular therapies with small molecules that enhance either CAR T-cell or CLL activation such as lenalidomide (40–42, 80–82) or CD40 agonists (78, 83) may improve therapeutic responses in CLL. Future investigation is needed to understand how we can optimize CAR design to improve costimulation as well as understand how combination therapies may improve responses in CLL.

## Supplementary Material

Supplemental Figures 1-4, Tables 1-3 Supplemental Figure 1: An overview of study design choices. Supplemental Figure 2: IL-2 Supplementation Enhances the APC phenotype of CLL cells. Supplemental Figure 3: Exogenous Co-stimulation Improves Second-generation CAR T cell Activation. Supplemental Figure 4: aCLL Stimulation Shows Inconsistent Cytokine Production After a 6hr Incubation. Supplemental Table 1: Full Antibody List. Supplemental Table 2: CLL Donor List. Supplemental Table 3: Full list of adjusted P-values from Holm-Sidak multiple comparisons.
