# Supplementary material for: Enhanced Costimulatory Signaling Improves CAR T-cell Effector Responses in CLL
Source: Cancer Res Commun. 2022 Sep 30;2(9):1089–103. doi: 10.1158/2767-9764.CRC-22-0200 (PMC10010331; doi:10.1158/2767-9764.CRC-22-0200)
Supplement: Supplemental Figures 1-4, Tables 1-3 — Supplemental Figure 1: An overview of study design choices. Supplemental Figure 2: IL-2 Supplementation Enhances the APC phenotype of CLL cells. Supplemental Figure 3: Exogenous Co-stimulation Improves Second-generation CAR T cell Activation. Supplemental Figure 4: aCLL Stimulation Shows Inconsistent Cytokine Production After a 6hr Incubation. Supplemental Table 1: Full Antibody List. Supplemental Table 2: CLL Donor List. Supplemental Table 3: Full list of adjusted P-values from Holm-Sidak multiple comparisons. [file crc-22-0200-s01.pdf]

Supplementary Information

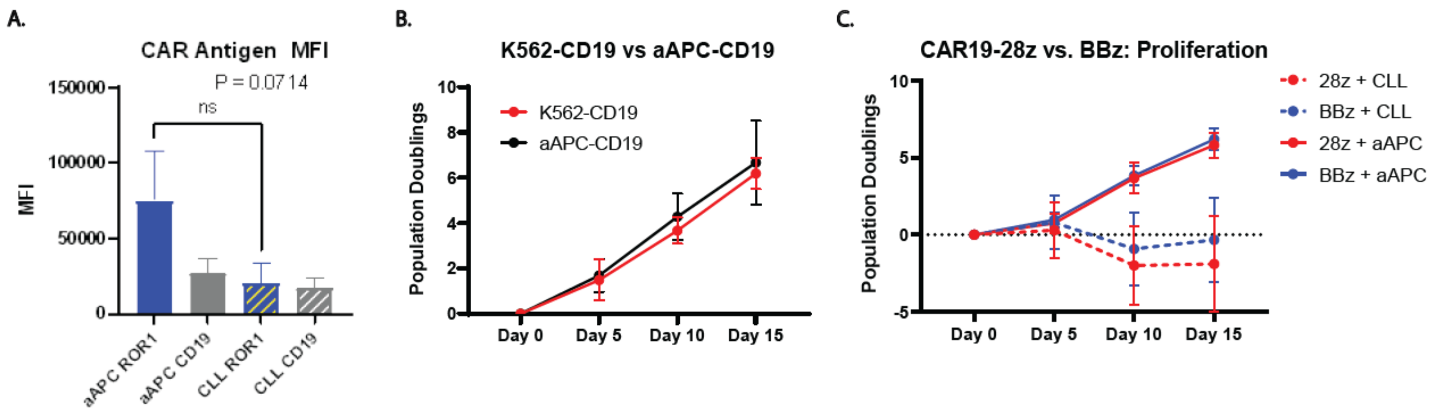

Supplemental Figure 1

Supplemental Figure 1: An overview of study design choices. (A) Depiction of the Mean Fluorescence Intensity (MFI) of the CAR target antigens ROR1 and CD19 on the aAPCs used in this study compared to the primary CLL cells. **(B)** Comparison of the proliferation of CAR19 T cells stimulated with either K562 cells expressing only CD19 or the aAPC cell line expressing CD19, CD86, and 4-1BBL. No significant difference in proliferation is observed over a 15-day re-stimulation assay. **(C)** Comparison of the proliferation of CD28-signaling (CAR19-28z) and 4-1BB-signaling (CAR19-BBz) CAR T cells stimulated with aAPCs or CLL cells over the 15-day re-stimulation assay. There is no difference in proliferation between the 28z- and BBz-signaling CARs. Panels A and B were analyzed using the multiple Mann-Whitney test with Holm-Sidak correction. Panel C was analyzed using the multiple Wilcoxon matched-pairs signed rank test. Panel A: n = 5 experiments. Panel B: n = 2 experiments, 3 T cell donors. Panel C: n = 2 experiments, 2 T cell donors, 6 CLL Donors = [4121, 4371, 4394, 4448, 4634, 6253].

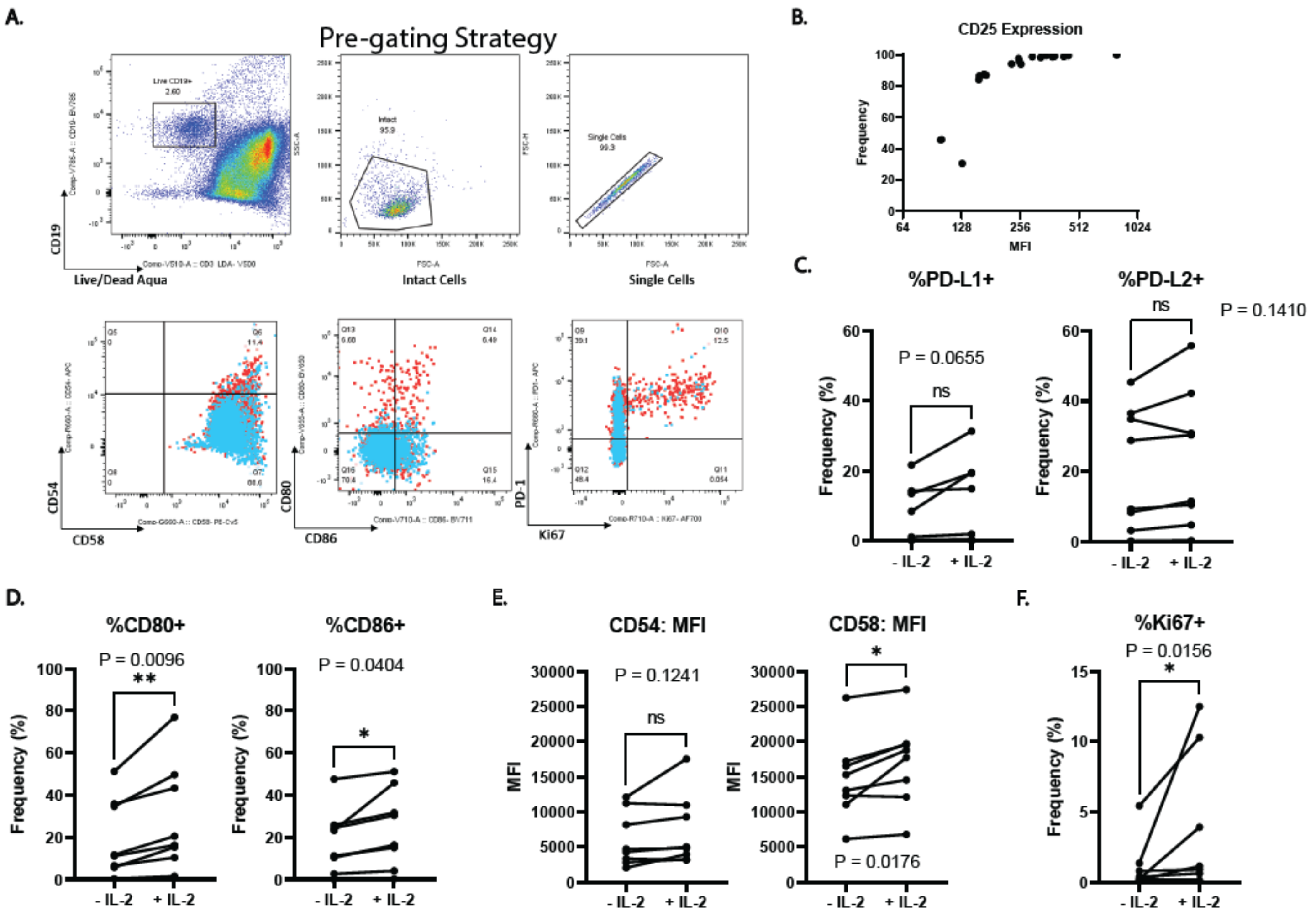

Supplemental Figure 2

Supplemental Figure 2: IL-2 Supplementation Enhances the APC phenotype of CLL cells. (A) Gating schematic for analysis of CLL cells. First, Live, CD19+ cells were selected, followed by intact and single cell gates (*left*). Gates were set using fluorescence-minus-one controls. Overlay flow plots showing changes in expression of CD54/CD58, CD80/CD86, and PD-1/Ki67 are shown on the *right*. Blue samples are resting CLL and red samples are CLL cells supplemented with 100U/ml IL-2. Flow was performed after 5 days. **(B)** CD25 expression on a panel of 20 primary CLL donors. Frequency of expression is given on the y-axis and MFI is given on the x-axis. **(C)** IL-2 supplementation did not significantly increase the frequency of PD-L1+ or PD-L2+ CLL cells. **(D)** Expression of costimulatory molecules CD80 and CD86 were increased with IL-2 supplementation. **(E)** Expression level of adhesion molecules CD54 and CD58 after 5 days either resting or with 100U/ml IL-2. **(F)** Expression of Ki67 as a readout of proliferation both with and without 100U/ml IL-2 addition. Panels B, C (middle, right), and D were analyzed using a two-tailed paired t test. Panel C (left) and E were analyzed using a two-tailed Wilcoxon matched-pairs signed rank test. (n = 2 experiments, 7 CLL donors: [2656, 2699, 3452, 4077, 4107, 4448, 4634], * $P<0.05$, ** $P<0.01$).

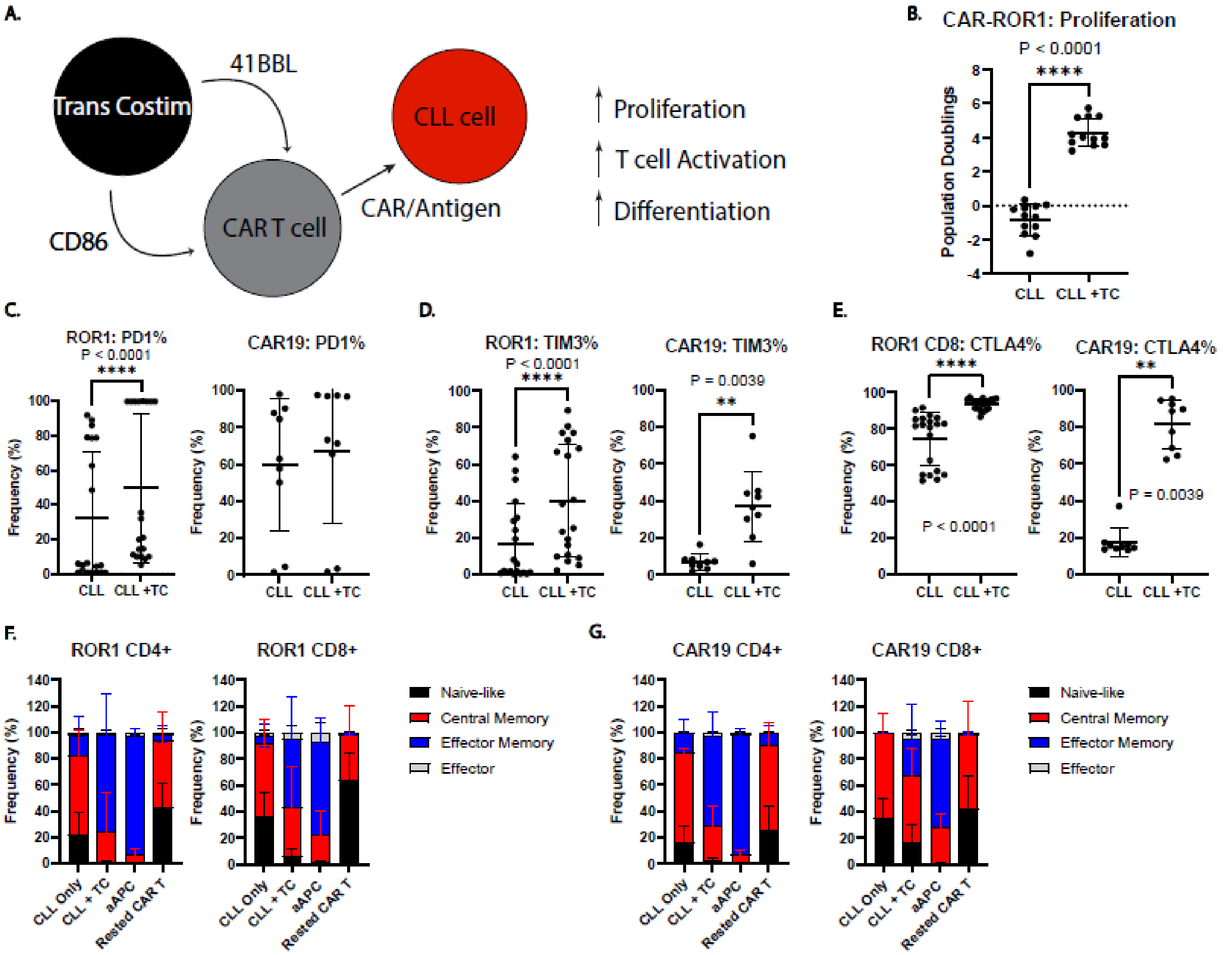

Supplemental Figure 3

Supplemental Figure 3: Exogenous Co-stimulation Improves Second-generation CAR T cell Activation. (A) Schematic depicting the trans-co-stimulation setup. CAR T cells are placed in culture with K562 cells expressing CD86 and 41BBL but no CAR target antigen. CAR T cells are then stimulated with primary CLL cells. Exogenous co-stimulation is therefore provided *in trans* to antigenic stimulation. **(B)** Proliferation of CAR ROR1 T cells after one stimulation with either CLL cells alone or CLL cells and trans-co-stimulation (TC). Proliferation is given as population doublings. **(C)** Frequency of PD-1+ CD8+ CAR ROR1 T cells (*left*) and CAR19 T cells (*right*) after one stimulation with CLL cells vs CLL and trans-co-stimulation. **(D)** Frequency of TIM3+ CD8+ CAR ROR1 T cells (*left*) and CAR19 T cells (*right*) after one stimulation with CLL cells vs CLL and trans-co-stimulation. **(E)** Frequency of CTLA4+ CD8+ CAR ROR1 T cells (*left*) and CAR19 T cells (*right*) after one stimulation with CLL cells vs CLL and trans-co-stimulation. **(F)** Quantification of the frequencies of the differentiation state of CAR ROR1 T cells defined as follows: *Effector:* CD27-CD45RO-. *Effector Memory:* CD27-CD45RO+. *Central Memory:* CD27+CD45RO+. *Naïve-like:* CD27+CD45RO-. CD4+ CAR-ROR1 T cells are depicted on the left and CD8+ CAR-ROR1 T cells are depicted on the right. Data were generated on day 5 after stimulation with CLL cells +/- TC. **(G)** Quantification of the frequencies of the differentiation state of CAR19 T cells after a five-day CLL stimulation +/- TC. Panels B, C (right), F (right), and G were analyzed using a two-tailed paired t test. Panels C (left), D, E, and F (left) were analyzed using the two-tailed Wilcoxon matched-pairs signed rank test. CAR ROR1: n = 4 experiments, 6 CLL Donors: [2722, 4077, 4107, 4394, 4448, 4634]. CAR19: n = 2 experiments, 6 CLL Donors: [2722, 4077, 4107, 4394, 4448, 4634], (**$P<0.01$, ****$P<0.0001$).

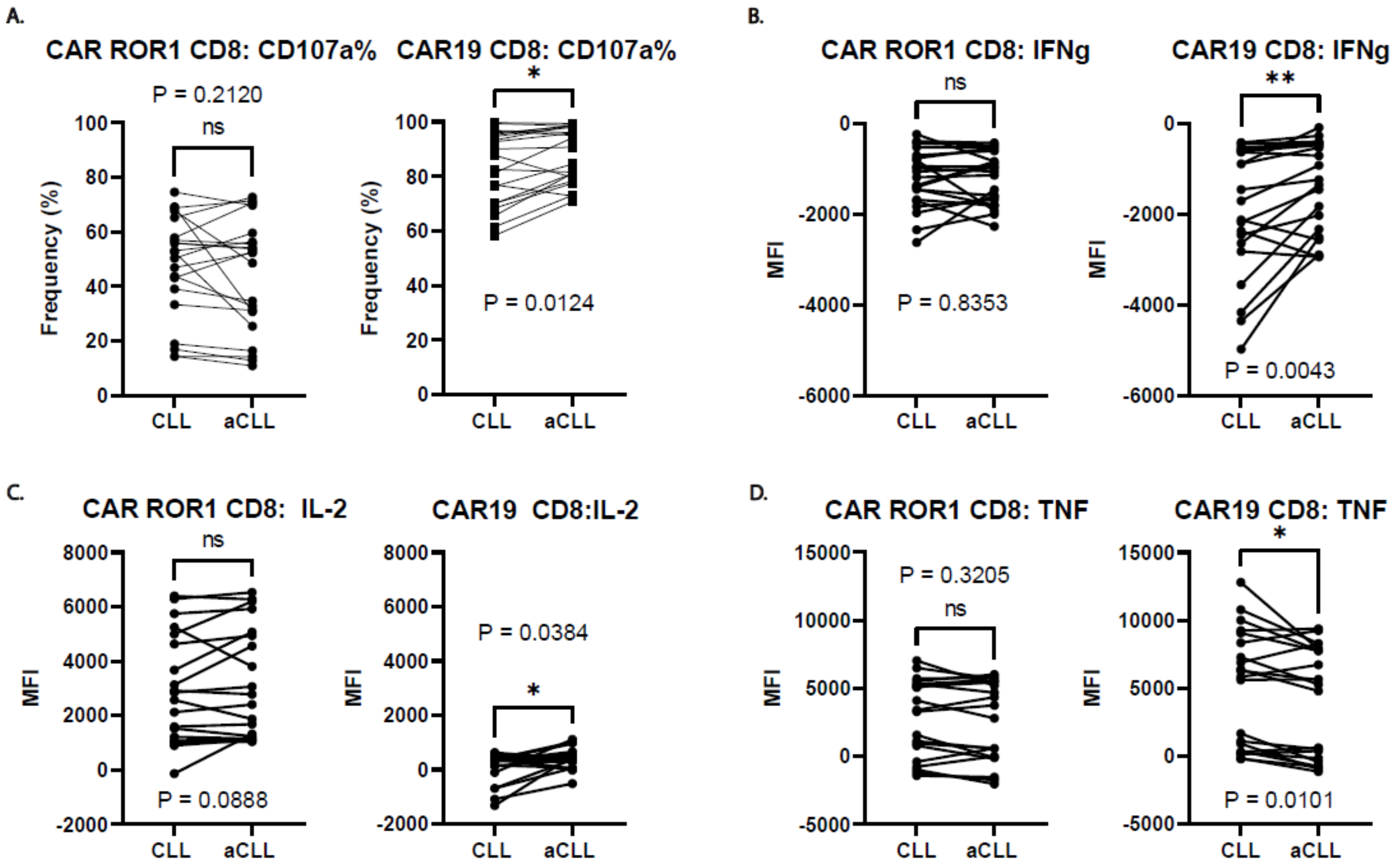

Supplemental Figure 4

Supplemental Figure 4: aCLL Stimulation Shows Inconsistent Cytokine Production After a 6hr Incubation. **(**A**)** Frequency of CD107a on CAR-ROR1+CD8+ (left) and CAR19+CD8+ (right) T cells following a 6hr stimulation with either un-activated CLL cells or aCLL cells. **(B)** MFI of IFNγ on CD8+ CAR ROR1 (*left*) and CAR19 (*right*) T cells following a 6hr incubation with either un-activated CLL cells or aCLL cells. **(C)** MFI of IL-2 on CD8+ CAR ROR1 (*left*) and CAR19 (*right*) T cells following a 6hr incubation with either un-activated CLL cells or aCLL cells. **(D)** MFI of TNFα on CD8+ CAR ROR1 (*left*) and CAR19 (*right*) T cells following a 6hr incubation with either un-activated CLL cells or aCLL cells. Panel A (right) Panel B (right), Panel C, and Panel D were analyzed using the two-tailed Wilcoxon matched-pairs signed rank test. Panel A (left) and Panel B (left) were analyzed using a two-tailed paired t test. (n = 2 experiments, 7 CLL donors: [2655, 2656, 4121, 4405, 4567, 4625, 4684], * P<0.05, **P<0.01).

Antibody Specificity	Clone Identifier	Conjugate	Supplier	Catalogue Number	RRID
Annexin V	-	APC	BD Biosciences	550474	AB_2921283
B7-H4	MIH43	BV421	BD Biosciences	562786	AB_2737793
CD138	MI15	PerCP-Cy5.5	BD Biosciences	564605	AB_2738862
CD14	MφP9	APC-H7	BD Biosciences	641394	AB_1645725
CD14	M5E2	V500	BD Biosciences	561391	AB_10611856
CD19	HIB19	V500	BD Biosciences	561121	AB_10562391
CD19	HIB19	BV785	BioLegend	302240	AB_2563442
CD19	V CD19.11	PE-Cy5	BioLegend	302210	AB_314240
CD200	325516	APC	R and D Systems	FAB27241A	AB_1061605
CD200R	OX-108	PE-Dazzle	BioLegend	329310	AB_2565526
CD25	2A3	PE	BD Biosciences	341009	AB_400203
CD27	O323	BV711	BioLegend	302833	AB_11219201
CD3	SK7	APC-H7	BD Biosciences	560176	AB_1645475
CD3	SP34-2	V500	BD Biosciences	560770	AB_1937322
CD3	UCHT1	BV570	BioLegend	300436	AB_2562124
CD4	OKT4	BV785	BioLegend	317441	AB_2561365
CD40	5C3	BV421	BD Biosciences	563396	AB_2738180
CD45	HI30	BV605	BioLegend	304042	AB_2562106
CD45RO	UCHL1	BV605	BioLegend	304237	AB_2562143
CD47	B6H12	FITC	BD Biosciences	556045	AB_396316
CD5	III 518	PE-Cy7	BioLegend	300622	AB_2275812
CD54	HA58	APC	BD Biosciences	559771	AB_398667
CD58	1C3	PE-Cy5	BD Biosciences	551399	AB_394183
CD68	Y1/82A	BV711	BD Biosciences	565594	AB_2739297
CD70	Ki-24	FITC	BD Biosciences	555834	AB_396157
CD8a	RPA-T8	BV650	BioLegend	301041	AB_11125174
CD80	L307.4	BV650	BD Biosciences	564158	AB_2738630
CD80	L307.4	BV786	BD Biosciences	564159	AB_2738631
CD86	IT2.2	BV711	BioLegend	305440	AB_2565835
CD86	IT2.2	BV785	BioLegend	305442	AB_2616794
EOMES	WD1928	FITC	ThermoFisher Scientific	11-4877-42	AB_2572499
Galectin-9	9M1-3	PerCP-Cy5.5	BioLegend	348910	AB_2562688
HLA-DR	G46-6	APC-R700	BD Biosciences	565127	AB_2732055
ICOSL	2D3/B7-H2	PE-CF594	BD Biosciences	564277	AB_2738726
IL-2	5344.111	PE-CF594	BD Biosciences	562384	AB_11154601
IFNγ	4S.B3	BV570	BioLegend	502534	AB_2563880
IFNγ	4S.B3	BUV737	BD Biosciences	612845	AB_2870167
Ki-67	B56	AF700	BD Biosciences	561277	AB_10611571
Granzyme B	GB11	PE-Cy5.5	ThermoFisher Scientific	GRB18	AB_2536541
LAG3	3DS223H	PE-Cy7	ThermoFisher Scientific	25-2239-42	AB_2573430
CTLA-4	BNI3	PE-Cy5	BD Biosciences	555854	AB_396177
OX40L	159403	PE	R and D Systems	FAB10541P	AB_10920764
PD-1	EH12.2H7	BV421	BioLegend	329920	AB_10960742
PD-1	EH12.2H7	APC	BioLegend	329908	AB_940475
PD-L1	29E.2A3	BV421	BioLegend	329714	AB_2563852
PD-L2	24F.10C12	PE	BioLegend	329606	AB_1089019
Perforin	B-D48	BV421	BioLegend	353307	AB_11149688
ROR1	2A2	PE-Cy7	BioLegend	357808	AB_2563276
TIM-3	F38-2E2	PE	BioLegend	345006	AB_2116576
TNFα	MAb11	AF700	BioLegend	502928	AB_2561315
7-AAD	-	-	BD Biosciences	559925	AB_2869266
Anti-FMC63	Y45	PE	ACRO Biosystems	FM3-HPY53	AB_2921284

Supplemental Table 1: Full Antibody List. List of all the antibodies used in this study including clone, supplier, conjugate, and catalogue number.

CLL Donor ID	Age (yrs)	Sex	Rai Status	Cytogenetics	Status, Time of Collection
1993	71.4	M	1	-	Refractory
2655	56.3	M	0	-	Untreated
2656	69.4	F	0	-	Untreated
2699	54.6	M	N/A	46, XY[19]	N/A
2722	65.2	M	0	-	Untreated
2761	51.3	M	4	-	Untreated
2771	57.1	M	N/A	-	Relapsed, Untreated
3416	77.7	M	N/A	-	Untreated
3431	53.5	F	0	46,XX,del(11)(q22q23)[13]/46,XX[7]	Untreated
3452	80.5	M	2	46,XY[3]	Relapsed, Bendamustine
3445	54.2	M	N/A	-	PR, Rituximab/Steroids
3507	68.8	M	1	-	Untreated
3578	54.3	M	1	-	Untreated
3935	78.5	F	1	del(13q14.3)	Untreated
3955	88	F	3	-	N/A
4045	68.8	M	0	del(13)	Untreated
4048	89.4	M	0	del(13q14.3), del(11q22_ATM)	Untreated
4077	72.4	M	N/A	46,X,-Y,+15[4]/45,X,-Y,del(13)(q12q14)[3]/45~46,XY,-Y,t(2;11)(p13;q22.3),del(13)(q12q14)[cp6]/46,XY[4]	PR, Ibrutinib
4107	59.8	F	N/A	-	PR, Ibrutinib
4121	58.1	M	0	del(13q)	Untreated
4129	43.7	M	2	-	Untreated
4167	77.5	M	0	-	Monthly IvIg
4227	48.5	F	1	del(p17)_p53,del(1q3)	Untreated
4265	64.5	M	3	-	Untreated
4276	72.4	F	2	-	Refractory
4288	46.5	M	1	del(13q14.3)	Refractory
4371	70.4	M	N/A	47,XY,t(3;20)(p21;q11.2),+12,add(18)(q21)[2]	Refractory
4394	54.4	F	2	47,XX,del(1)(q42q43),+21[cp15]/47,idem,add(1)(q42)[2]/46,XX[3]	PR, Ibrutinib
4405	68.6	F	3	45,X,-X[4]/46,XX[16]	Untreated
4419	54.7	F	3	-	Untreated
4427	58.7	F	N/A	-	Untreated
4430	88.7	F	1	-	Untreated
4444	59	M	1	-	Untreated
4448	46.8	M	N/A	-	PR, Ibrutinib
4487	49.7	F	2	46,XX,del(13)(q12q21)[8]/46,XX[7]	Untreated
4490	50.7	M	2	46~47,XY,del(6)(q13q25),?dic(7;21)(q31;p13),add(11)(q13),del(13)(q12q14),+2mar,inc[cp4]/46,XY[3]	Untreated
4516	87.3	F	3	-	Untreated
4532	58.8	F	2	46,XX,der(17)t(17;?21)(p13;?q11.2)[10]	Untreated
4556	65.9	F	3	del(p17)_p53	Untreated
4567	81.9	M	3	46,XY[3]	Untreated
4599	50.8	M	2	46~47,XY,del(6)(q13q25),?dic(7;21)(q31;p13),add(11)(q13),del(13)(q12q14),+2mar,inc[cp4]/46,XY[3]	Untreated
4625	66	F	3	-	Untreated
4634	70.8	M	N/A	-	Refractory, Ibrutinib
4665	67.3	M	0	-	Untreated
4794	50.3	M	2	46,XY,t(1;17;13)(p13;p11.2;q12)[11]/46,XY[12]	Untreated
5013	65.8	F	0	-	Untreated
5071	72.1	M	N/A	4,XY,-5,t(5;22)(p15;q12), del(10)(q23),i(17)(q10),-18,-19,+mar[9]/46,XY[4], del(p53)	N/A
5083	58.2	F	N/A	-	Untreated
5108	76.2	M	1	47,XY,+12[3]/46,XY[2]	Untreated
5131	65	F	N/A	-	Untreated
5267	69.3	F	N/A	-	N/A
5529	72.6	M	N/A	-	Untreated
5574	68.6	M	4	45,XY,del(5)(q?13q?22),der(17;18)(q10;q10)[3]/45,idem,add(7)(q36),add(22)(q13)[15]/46,XY[2]	Relapsed
5597	69.9	M	1	47,XY,+12[5]/46,XY[15]	Untreated
5786	68.2	F	1	47,XX,+12	Untreated
5798	64.1	M	1	47,XY, +12	Untreated
5891	49	M	0	46,XY,t(4;12)(q25;p12)[5]/47,idem,+3[15]	Untreated
5895	78.5	M	2	-	Untreated
5963	68.7	F	1	47,XX,+12[14]/46,XX[1]	Untreated
6127	68.7	F	0	-	Untreated
6197	69.2	F	0	-	Untreated
6253	55.8	M	N/A	-	Untreated
6342	61.3	M	1	-	Untreated
6345	59.8	M	N/A	-	Untreated

Supplemental Table 2

Supplemental Table 2: CLL Donor List. List of CLL donors used in this study. Phenotypic information including patient age, sex, Rai Status at the time of sample collection, cytogenetic information, and treatment at the time of sample collection. Information on treatments prior to collection that are no longer ongoing are not available.

Figure	Comparison	Un-adjusted P-value	Holm-Sidak Adjusted P-value	Number of Comparisons
1C	CLL+CARROR1 vs aAPC+CARROR1 Day 5	0.002451	0.009768	3
	CLL+CARROR1 vs aAPC+CARROR1 Day 10	0.002451	0.009768	
	CLL+CARROR1 vs aAPC+CARROR1 Day 15	0.002451	0.009768	
	CLL+CAR19 vs aAPC+CAR19 D5	0.236963	0.417774	3
	CLL+CAR19 vs aAPC+CAR19 D10	0.002799	0.008375	
	CLL+CAR19 vs aAPC+CAR19 D15	0.000099	0.000396	
1D (left)	CAR ROR1 CLL vs aAPC Day 5	0.002451	0.007335	3
	CAR ROR1 CLL vs aAPC Day 10	0.002451	0.007335	
	CAR ROR1 CLL vs aAPC Day 15	0.002451	0.007335	
1D (right)	CAR19 CLL vs aAPC Day 5	0.000050	0.000099	3
	CAR19 CLL vs aAPC Day 10	0.000226	0.000226	
	CAR19 CLL vs aAPC Day 15	0.000025	0.000074	
1E (left)	CAR ROR1 CLL vs aAPC Day 5	0.002451	0.007335	3
	CAR ROR1 CLL vs aAPC Day 10	0.002451	0.007335	
	CAR ROR1 CLL vs aAPC Day 15	0.036765	0.036765	
1E (right)	CAR19 CLL vs aAPC Day 5	0.000050	0.000149	3
	CAR19 CLL vs aAPC Day 10	0.365669	0. 365669	
	CAR19 CLL vs aAPC Day 15	0.021909	0.043337	
1F (left)	CAR ROR1 CLL vs aAPC Day 5	0.002451	0.007335	3
	CAR ROR1 CLL vs aAPC Day 10	0.056373	0.056373	
	CAR ROR1 CLL vs aAPC Day 15	0.002451	0.007335	
1F (right)	CAR19 CLL vs aAPC Day 5	0.746761	0.935870	3
	CAR19 CLL vs aAPC Day 10	0.844946	0.935870	
	CAR19 CLL vs aAPC Day 15	0.376935	0.758120	
1G (left)	CAR ROR1 CLL vs aAPC Day 5	0.004902	0.014634	3
	CAR ROR1 CLL vs aAPC Day 10	0.352941	0.581315	
	CAR ROR1 CLL vs aAPC Day 15	0.654412	0.654412	
1G (right)	CAR19 CLL vs aAPC Day 5	0.280094	0.297281	3
	CAR19 CLL vs aAPC Day 10	0.161717	0.297281	
	CAR19 CLL vs aAPC Day 15	0.009872	0.029326	
1H (ROR1)	10:1	0.004396	0.021786	5
	3:1	0.015385	0.060133	
	1:1	0.043956	0.085980	
	1:3	0.214286	0.214286	
	1:10	0.016667	0.060133	
1H (CAR19)	10:1	0.008081	0.039756	5
	3:1	0.048485	0.138516	
	1:1	0.214141	0.382426	
	1:3	0.028283	0.108422	
	1:10	0.214141	0.382426	
2D	A vs CA IFNg	0.004428	0.013225	3
	A vs CA IL-2	0.245505	0.358963	
	A vs CA TNF	0.199352	0.358963	
	A vs CCA IFNg	0.000178	0.000535	3
	A vs CCA IL-2	0.224063	0.397922	
	A vs CCA TNF	0.378124	0.397922S	
2E	CCC vs CCA CD4	0.002165	0.004324	2
	CCC vs CCA CD8	0.002165	0.004324	

	CCC vs CAA CD4	0.002165	0.004324	2
	CCC vs CAA CD8	0.041126	0.041126	
3B	4:0 vs 0:4 Day 5	0.555664	0.555664	3
	4:0 vs 0:4 Day 10	0.000977	0.001952	
	4:0 vs 0:4 Day 15	0.000488	0.001464	
	3:1 vs 0:4 Day 5	0.176270	0.176270	3
	3:1 vs 0:4 Day 10	0.026855	0.052990	
	3:1 vs 0:4 Day 15	0.000488	0.001464	
	2:2 vs 0:4 Day 5	0.203613	0.203613	3
	2:2 vs 0:4 Day 10	0.000977	0.001952	
	2:2 vs 0:4 Day 15	0.000488	0.001464	
	1:3 vs 0:4 Day 5	0.129395	0.129395	3
	1:3 vs 0:4 Day 10	0.000488	0.001464	
	1:3 vs 0:4 Day 15	0.000488	0.001464	
5B (left)	CLL+CARROR1 vs ivLNCLL+CARROR1 D5	<0.000001	<0.000001	3
	CLL+CARROR1 vs ivLNCLL+CARROR1 D10	<0.000001	<0.000001	
	CLL+CARROR1 vs ivLNCLL+CARROR1 D15	<0.000001	<0.000001	
5B (right)	CLL+CAR19 vs aCLL+CAR19 D5	0.000309	0.000309	3
	CLL+CAR19 vs aCLL+CAR19 D10	0.000002	0.000006	
	CLL+CAR19 vs aCLL+CAR19 D15	0.000132	0.000263	
5D (left)	aCLL+CARROR1 vs aCLL+CARROR1 10:1	0.719238	0.719238	5
	aCLL+CARROR1 vs aCLL+CARROR1 3:1	0.000977	0.003901	
	aCLL+CARROR1 vs aCLL+CARROR1 1:1	0.000977	0.003901	
	aCLL+CARROR1 vs aCLL+CARROR1 1:3	0.000488	0.002439	
	aCLL+CARROR1 vs aCLL+CARROR1 1:10	0.001953	0.003902	
5E (left)	aCLL+CAR19 vs aCLL+ CAR19 10:1	0.007813	0.038457	5
	aCLL+ CAR19 vs aCLL+ CAR19 3:1	0.023438	0.046146	
	aCLL+ CAR19 vs aCLL+ CAR19 1:1	0.007813	0.038457	
	aCLL+ CAR19 vs aCLL+ CAR19 1:3	0.015625	0.046146	
	aCLL+ CAR19 vs aCLL+CAR19 1:10	0.015625	0.046146	

Supplemental Table 3: Full list of adjusted P-values from Holm-Sidak multiple comparisons.
